# Ventricular dyssynchrony assessment using ultra-high frequency ECG technique

**DOI:** 10.1007/s10840-017-0268-0

**Published:** 2017-07-10

**Authors:** Pavel Jurak, Josef Halamek, Jaroslav Meluzin, Filip Plesinger, Tereza Postranecka, Jolana Lipoldova, Miroslav Novak, Vlastimil Vondra, Ivo Viscor, Ladislav Soukup, Petr Klimes, Petr Vesely, Josef Sumbera, Karel Zeman, Roshini S. Asirvatham, Jason Tri, Samuel J. Asirvatham, Pavel Leinveber

**Affiliations:** 1 Institute of Scientific Instruments of the Czech Academy of Sciences, Brno, Czech Republic; 2 International Clinical Research Center, St. Anne’s University Hospital, Brno, Czech Republic; 3 1st Department of Internal Medicine-Cardioangiology, St. Anne’s University Hospital, Masaryk University, Brno, Czech Republic; 4 Student Scholar Program, Mayo Clinic, Rochester, MN USA; 5 Division of Cardiovascular Diseases, Department of Internal Medicine, Mayo Clinic, Rochester, MN USA; 6 Department of Pediatrics and Adolescent Medicine, Mayo Clinic, Rochester, MN USA

**Keywords:** Ventricular dyssynchrony, Cardiac resynchronization therapy, High-frequency electrocardiography, Left bundle branch block, Depolarization

## Abstract

**Purpose:**

The aim of this proof-of-concept study is to introduce new high-dynamic ECG technique with potential to detect temporal-spatial distribution of ventricular electrical depolarization and to assess the level of ventricular dyssynchrony.

**Methods:**

5-kHz 12-lead ECG data was collected. The amplitude envelopes of the QRS were computed in an ultra-high frequency band of 500–1000 Hz and were averaged (UHFQRS). UHFQRS V lead maps were compiled, and numerical descriptor identifying ventricular dyssynchrony (UHFDYS) was detected.

**Results:**

An electrical UHFQRS maps describe the ventricular dyssynchrony distribution in resolution of milliseconds and correlate with strain rate results obtained by speckle tracking echocardiography. The effect of biventricular stimulation is demonstrated by the UHFQRS morphology and by the UHFDYS descriptor in selected examples.

**Conclusions:**

UHFQRS offers a new and simple technique for assessing electrical activation patterns in ventricular dyssynchrony with a temporal-spatial resolution that cannot be obtained by processing standard surface ECG. The main clinical potential of UHFQRS lies in the identification of differences in electrical activation among CRT candidates and detection of improvements in electrical synchrony in patients with biventricular pacing.

**Electronic supplementary material:**

The online version of this article (doi:10.1007/s10840-017-0268-0) contains supplementary material, which is available to authorized users.

## Introduction

Pathological changes in the structure of cardiac ventricles often manifest themselves in electrical activity. The clinical 12-lead ECG-based criteria for left ventricle (LV) dyssynchrony quantification and cardiac resynchronization therapy (CRT) implementation are based primarily on the duration and morphology of the QRS complex [1–3]. Unfortunately, QRS descriptors still do not appear to be effective tools for the diagnosis of LV dyssynchrony [4–8].

Conventional ECG diagnostic information is usually limited to 100 Hz. Most QRS parameters including QRS duration or morphology use frequency band below 30 Hz. Broad-band ECG measured by high-frequency and high-resolution monitors provide more precise QRS morphology [9] and information not accessible by conventional ECG. In 1981, Goldberger et al. [10] reported the effect of myocardial infarction on low-voltage high frequency (HF, 150–250 Hz) potentials in the QRS complex (HFQRS, HF-ECG). Studies dedicated to this topic [10–12] have shown that HFQRS potential morphology and amplitude reduction are modified by coronary occlusion or infarct-induced ischemia. When compared to commonly used ECG parameters such as the duration of the QRS complex, ST-segment abnormalities, fragmented QRS, and upward and downward slopes of the QRS complex [9, 11], cardiac ischemic pathology is detected with higher sensitivity using HFQRS. However, the presented methodologies have focused predominantly on heart ischemia (single-lead HFQRS morphology), neglected the temporal-spatial properties (dyssynchrony), and analyzed ECG in a limited frequency range (up to 250 Hz) [12, 13].

Here, we introduce an ultra-high frequency electrocardiogram (UHF-ECG) technique. The higher frequency and the dynamic range allow for more accurate identification of the temporal distribution of electrical depolarization in a single lead. The mutual comparison of depolarization activation patterns in different ventricular segments (leads) and computation of depolarization inter-lead delays can be potentially interpreted as ventricular dyssynchrony.

## Methods

### Data acquisition and processing

The acquisition system BioSDA09 (M&I, Prague, CZ) was used to record ECG signals at 5-kHz with a dynamic range of 26 bits (3 nV resolution) and a frequency range of 2-kHz. Measurements were performed at the International Clinical Research Center at St. Anne’s University Hospital, Brno, Czech Republic. The UHF-ECG data was collected over 5–15 min in the resting supine position with a standard 12-lead ECG setup.

The UHF Solver and SignalPlant (ISI CAS, Brno, CZ) [14] software was specifically developed for UHF-ECG data processing. Stimulating peaks were removed from the signal in patients with pacemakers. QRS complexes were detected [15] and clustered into different QRS morphology categories using a robust multichannel approach capable of recognizing sinus QRS patterns as well as irregular patterns. This technique was used to focus the analysis primarily on the sinus (dominant) rhythm. The amplitude envelopes were computed in a frequency band of 500–1000 Hz using the Hilbert transform and were averaged with an R-wave trigger and smoothed in a 0–40 Hz passband (UHFQRS). The stimulation peaks detection and removal and QRS categorization are the crucial parts of UHF-ECG signal pre-processing. The precision of signal pre-processing substantially influences the signal-to-noise ratio in averaged UHFQRS envelopes.

**Fig. 1:**
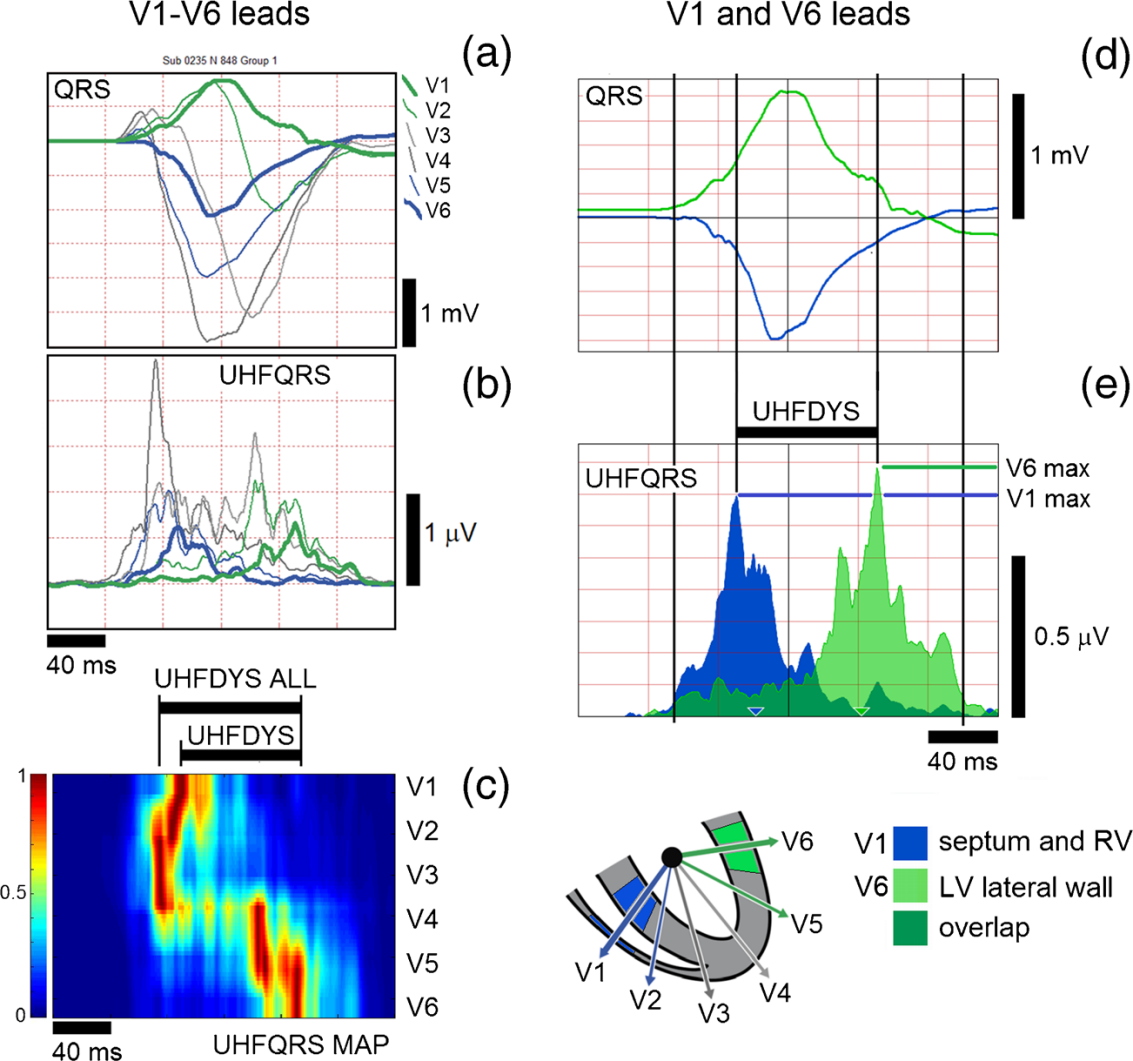
UHFQRS and UHFQRS maps, patient 1, LBBB. *Left panel*, V1–V6 leads. **a** Averaged QRS complexes (average from 848 heart beats). **b** Averaged UHF 500–1000 Hz amplitude envelopes (UHFQRS). **c** Normalized UHFQRS maps (maximum in each map row (lead) is normalized to 1 (*red*), minimum is normalized to 0 (*blue*). *Right panel*, V1 and V6 leads. **d** Averaged QRS. **e** Averaged UHFQRS, UHFDYS—difference in positions of UHFQRS maxima in leads V1 and V6, UHFDYS ALL—maximal difference in positions of UHFQRS maxima in all V leads. UHFDYS ALL represents maximal intraventricular dyssynchrony. The *blue* color corresponds to the V1 lead and, the *light green* color to the V6 lead, the *dark green* color represents the overlap of V1 and V6 depolarization

The shapes of averaged QRS complexes (Fig. 1a) of V leads can be compared to averaged UHFQRS envelopes (Fig. 1b) and UHFQRS maps (Fig. 1c). Each horizontal row of the map represents the normalized shape of UHFQRS for single V lead. The maximum in each map row (lead) is normalized to 1 (red), while the minimum is normalized to 0 (blue). Normalization standardizes the UHFQRS to the uniform amplitude range of 0–1. The parameter UHFDYS (Fig. 1e), defined by differences in the positions of maxima in leads V1 and V6, is especially helpful in the numerical identification of ventricular electrical dyssynchrony. The UHFDYS parameter in Fig. 1 means that peak depolarization in the RV and septum precedes peak depolarization in the LV lateral wall by 84 ms. This value is clearly identifiable from UHFQRS (Fig. 1b–e) but cannot be detected from standard (low-frequency) QRS (Fig. 1a, d). An example of a UHFQRS map (Fig. 1c) also demonstrates that dyssynchrony UHFDYS in V1 and V6 leads sometimes does not reflect the highest dyssynchrony. In this case, V2 and V6 dyssynchrony (Fig. 1c, UHFDYS ALL) is 96 ms. Therefore, both C and E images are important for presentation of the results.

Detailed description of UHF-ECG signal processing including comparison of different frequency ranges is available in the Electronic Supplementary Material.

### Patient selection

Initially, we have studied seven patients with characteristic QRS complex shape. Table 1 (subjects 1–7) presents their baseline clinical data. To assess predictive power of UHF-ECG-derived dyssynchrony for identification of responders to CRT, we started prospective 6-month follow-up study. The baseline data of first ten consecutive patients who completed that follow-up interval are demonstrated in Table 1 (patients 8–17). All patients were in sinus rhythm and did not have signs of cardiac decompensation. CRT patients were selected on the basis of European Society of Cardiology criteria for CRT [17]. Two patients were implanted even if their LVEF mildly exceeded the recommended guidelines cut-off value of 35%. All CRT subjects underwent UHF-ECG and standard transthoracic echocardiography within 24 h before CRT and at 6 months after CRT.

**Table 1:** Baseline patient characteristics with echocardiographic parameters

Pt.	Age	Gender	Diagnosis	AA medication	Conduction disturbances	QRS Strauss criteria	QRSd (ms)	LVEF^*^ (%)	ESV^*^ (ml)
1	78	M	CAD	Metoprolol	LBBB	0	191	25	–
2	53	F	DCM	**–**	LBBB	1	148	30	–
3	58	M	Heart transplant	–	RBBB	–	139	60	–
4	71	M	DCM	Bisoprolol	LBBB	1	190	25	–
5	28	M	WPW sy.	–	RSAP	–	105	56	–
6	66	M	DCM	Bisoprolol	LBBB	1	194	25	–
7	73	F	DCM	Metoprolol	–	0	193	25	–
8	45	M	DCM	–	LBBB	1	163	30	176
9	67	M	DCM	Bisoprolol	LBBB	1	191	32	183
10	65	F	DCM	–	LBBB	0	182	26	136
11	71	M	DCM	Bisoprolol	LBBB	1	192	38	102
12	78	M	VHD	Amiodarone	LBBB	1	178	23	176
13	76	M	CAD	Bisoprolol	LBBB	1	181	39	132
14	72	M	VHD	Bisoprolol	LBBB	0	158	25	141
15	78	M	CAD	Carvedilol	–	0	138	16	256
16	50	M	DCM	Carvedilol	LBBB	1	140	21	246
17	65	M	CAD + DCM	Nebivolol	LBBB	1	151	25	198

*Pt*. patient, *M/F* male/female, *AA* medication Antiarrhythmic medication, *QRSd* QRS duration, *LVEF* left ventricular ejection fraction, *ESV* end-systolic volume, *CAD* coronary artery disease, *DCM* dilated cardiomyopathy, *WPW* sy. Wolf-Parkinson-White syndrome, *VHD* valvular heart disease, *LBBB* left bundle branch block, *RBBB* right bundle branch block, *RSAP* right-sided accessory pathway

^*^Standard 2-dimensional echocardiography was used to obtain LV volumes and EF [16]

All subjects gave their informed consent to the investigation. This study was approved by the local ethics committee—The Ethics Committee at St. Anne’s Hospital, Brno, Czech Republic.

### UHFQRS physiology

**Fig. 2:**
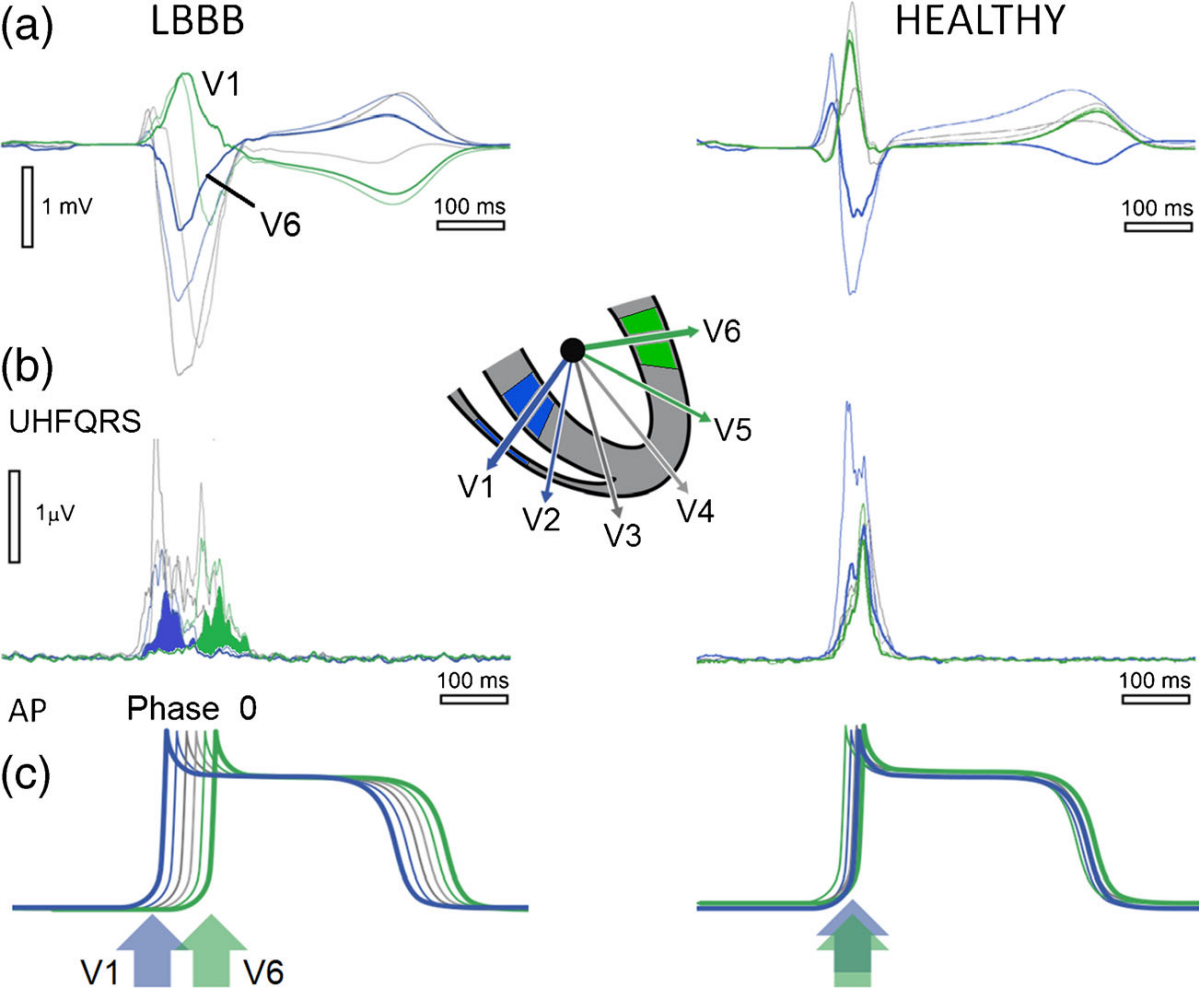
Physiological origin of UHFQRS electrical activation pattern in ventricular dyssynchrony. Patient 1 with delayed activation of LV lateral wall (LBBB) and subject with HEALTHY conduction system. **a** Averaged ECG, V1-V6 leads. **b** Averaged UHF amplitude envelopes 500–1000 Hz (UHFQRS). **c** Schematic interpretation of myocardial cell action potentials—AP. Blue—V1 lead—mostly interventricular septum and RV, green—V6 lead—mostly LV lateral wall

Fig. 2 demonstrates the physiological origin of UHF oscillations. The depolarization phase of myocardial cell action potentials (AP) serves as a main UHF transmitter (Fig. 2c). Steep cell membrane potential gradients caused by the sodium ion current change (Phase 0 of AP) represent a unique source of UHF oscillations. The mass of myocardium, especially the left ventricle, defines the main location of UHF transmitters. UHFQRS can be simply interpreted as a histogram of UHF oscillation distribution in time (horizontal time axis) and location (V leads). The UHF transmitters work synchronously in the healthy heart (Fig. 2b, HEALTHY). In a dyssynchronous left bundle branch block (LBBB) heart, the depolarization in the RV and septum (blue color in Fig. 2b, LBBB) precedes the depolarization in the LV lateral wall (green color).

### UHFQRS and two-dimensional speckle tracking echocardiography (STE)

**Fig. 3:**
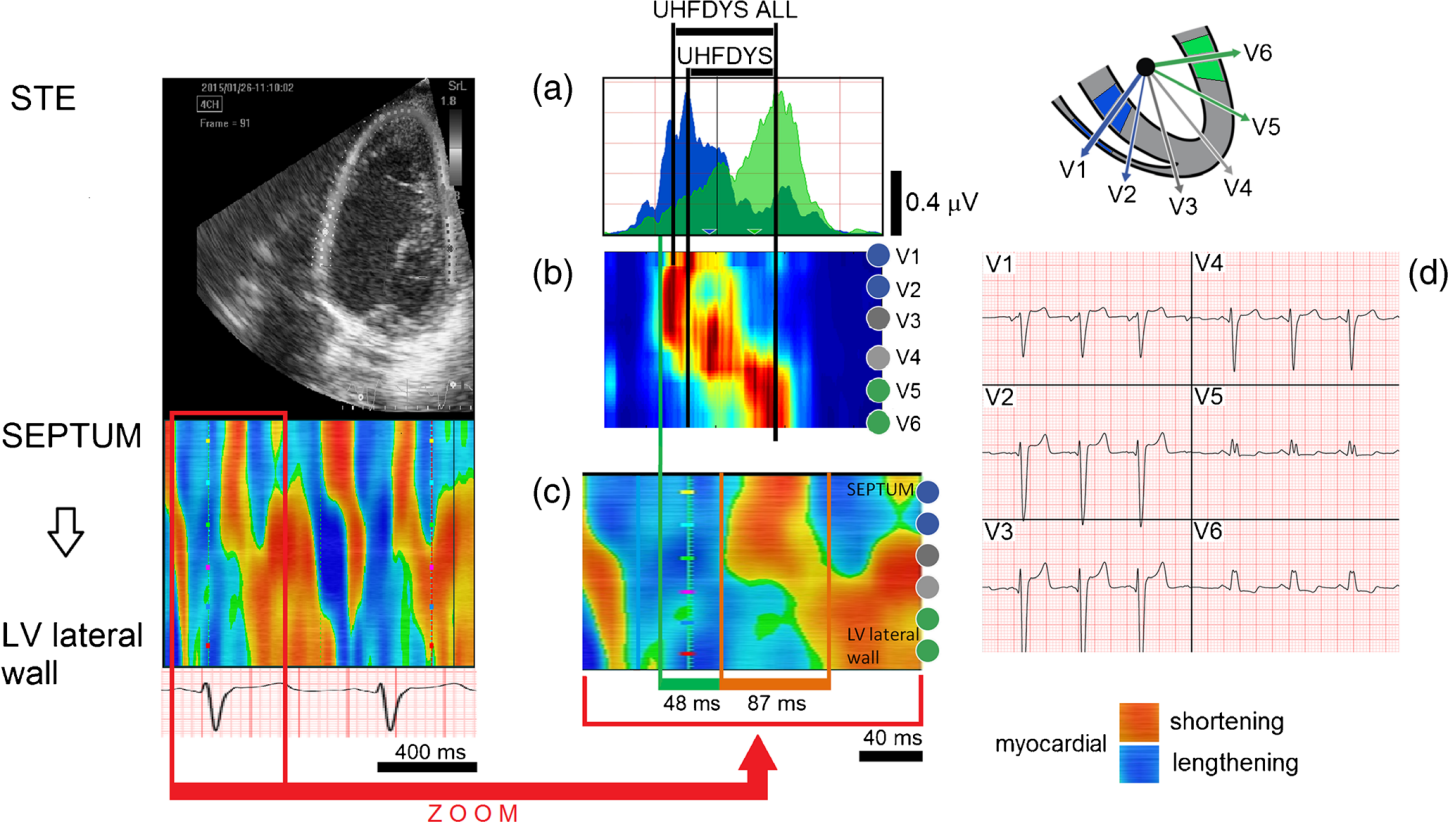
Electrical and mechanical dyssynchrony coupling demonstrated by UHFQRS and speckle tracking echocardiography (STE) in patient 2 suffering from LBBB. The figure compares the UHF electrical dyssynchrony and the mechanical dyssynchrony of the septum and LV lateral wall. Myocardial shortening is coded by the *orange/red* color and myocardial lengthening by the *blue* color. **a** UHFQRS, V1 (*blue*) and V6 (*green*) leads. **b** Normalized UHFQRS map. **c** Detail from STE map temporally synchronized with A and B. **d** V1-V6 ECG. UHFDYS and UHFDYS ALL electrical dyssynchrony are 61 and 74 ms, respectively,—*black horizontal bars*. **a** The time delay of mechanical motion between the onset of myocardial deformation of the middle septum and the middle lateral wall is 87 ms—*orange horizontal bar*. **c**. The green horizontal bar defines delay 48 ms between the first electrical UHF activation in V2 lead and onset of mechanical myocardial deformation of the middle septum

The main aim of cardiac resynchronization therapy is to improve mechanical synchrony [6]. STE was used to demonstrate the relationship of the electrical activation derived from the UHFQRS and the initiation of mechanical contraction (Fig. 3). STE is an angle-independent method that depicts myocardial deformation without the influence of extra-cardiac motion. Using the apical 4-chamber view, STE enables simultaneous detection of the onset of myocardial deformation of the septum and lateral wall. Fig. 3 demonstrates the match between electrical and mechanical activation and LV dyssynchrony in a patient suffering from LBBB.

The STE analysis software depicted the color-coded strain rate map from the basal, middle, and apical segments of the septum and left lateral wall (Fig. 3c). The onset of isovolumic deformation of each segment was defined by the first appearance (left margin) of the orange/red area at the electrocardiographic QRS complex region (red arrow). The mechanical STE septal-to-lateral asynchrony (Fig. 3c, orange horizontal bar, 87 ms) is analogical to the electrical UHFDYS (61 ms) and UHFDYS ALL (74 ms) asynchrony (Fig. 3a, black horizontal bars). Similarly, the course of electrical UHFQRS maps correspond to the course of the beginnings of myocardial deformations of the pertinent myocardial segments. Comparison of UHFQRS and STE has also provided a new parameter: the time delay between electrical and mechanical action onset (Fig. 3c, green horizontal bar). This time delay could define a physiological link between electrical activation and the onset of myocardial deformation in ventricles.

## Results

### Typical UHFQRS patterns—Normal, RBBB, LBBB, and RV pre-excitation

**Fig. 4:**
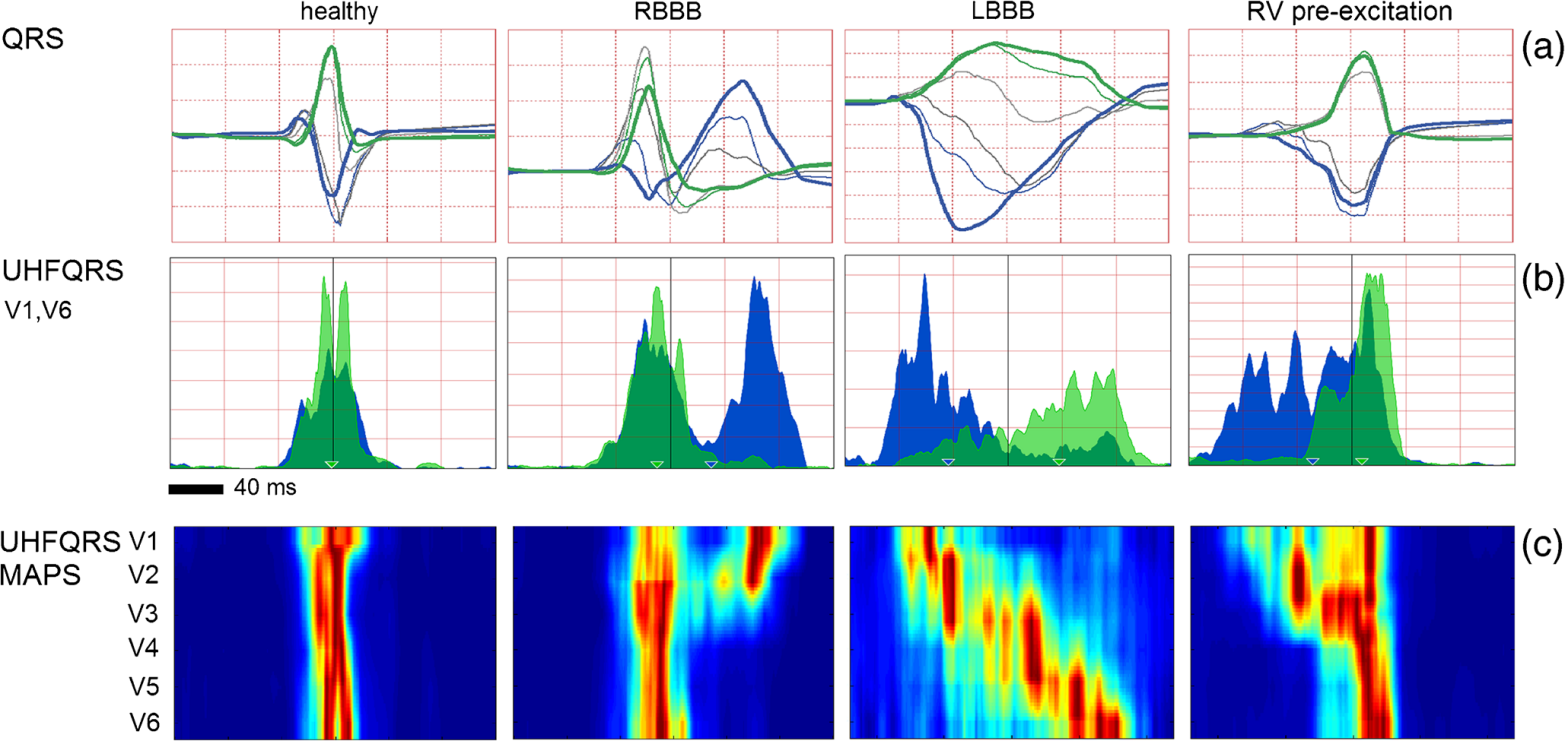
Examples of different ventricular electrical activation patterns. **a** Averaged QRS complexes, V leads. **b** Averaged UHFQRS of leads V1 (*blue*) and V6 (*green*). **c** UHFQRS maps. From left: healthy heart QRSd 81 ms, patient 3—RBBB, QRSd 139 ms, patient 4—LBBB, QRSd 190 ms, and patient 5 with WPW syndrome with right lateral accessory pathway QRSd 105 ms.

Figure 4 shows examples of typical UHFQRS pattern, from the left: a normal healthy heart, a patient with right bundle branch block (RBBB), a patient with LBBB, and a patient with WPW syndrome with RV pre-excitation. The UHFQRS maps (Fig. 4c) provide a detailed overview of the temporal-spatial distribution of depolarization computed from V leads. The UHFQRS maps demonstrate how the change in electrical depolarization synchrony can vary across different pathologies. Nevertheless, the description of dyssynchrony by UHFQRS in leads V1 and V6 (Fig. 4b) simplifies interpretation and allows for inter-subject computation of comparable numerical parameters.

### UHFQRS CRT response, LV pre-excitation

Table 2 demonstrates the first results of patients before and 6-month after CRT. The patients No. 8–14 represent responders with post-implantation LV ESV reduction of 10% or more and patients No. 15–17 non-responders [18]. In our preliminary analysis, UHF-ECG dyssynchrony with cut-off value of more than 50 ms identified correctly all responders and non-responders.

**Table 2:** Patient characteristics with DYS parameter before CRT and echocardiographic, QRSd, and DYS results 6 months after CRT

Pt.	DYS before CRT (ms)	6 months after CRT							
		LVEF^*^ (%)	LVEF change (%)	ESV^*^ (ml)	ESV change (%)	QRSd (ms)	QRSd change (ms)	DYS (ms)	DYS change (ms)
8	90	42	40	138	−22	100	−63	4	−86
9	101	38	19	137	−25	142	−49	8	−93
10	112	34	31	87	−36	137	−45	39	−73
11	122	46	21	70	−31	124	−68	−2	−124
12	97	34	48	155	−12	152	−26	35	−62
13	82	46	18	97	−27	145	−36	18	−64
14	53	37	48	108	−23	148	−10	−6	−59
15	28	11	−31	282	10	141	3	22	−6
16	39	20	−5	242	−2	128	−12	−5	−44
17	29	24	−4	188	−5	148	−3	−12	−41

*Pt*. patient, *QRSd* QRS duration, *LVEF* left ventricular ejection fraction, *ESV* end-systolic volume. Patients No.8–14 are responders to cardiac resynchronization therapy

^*^Standard 2-dimensional echocardiography was used to obtain LV volumes and EF [16]

**Fig. 5:**
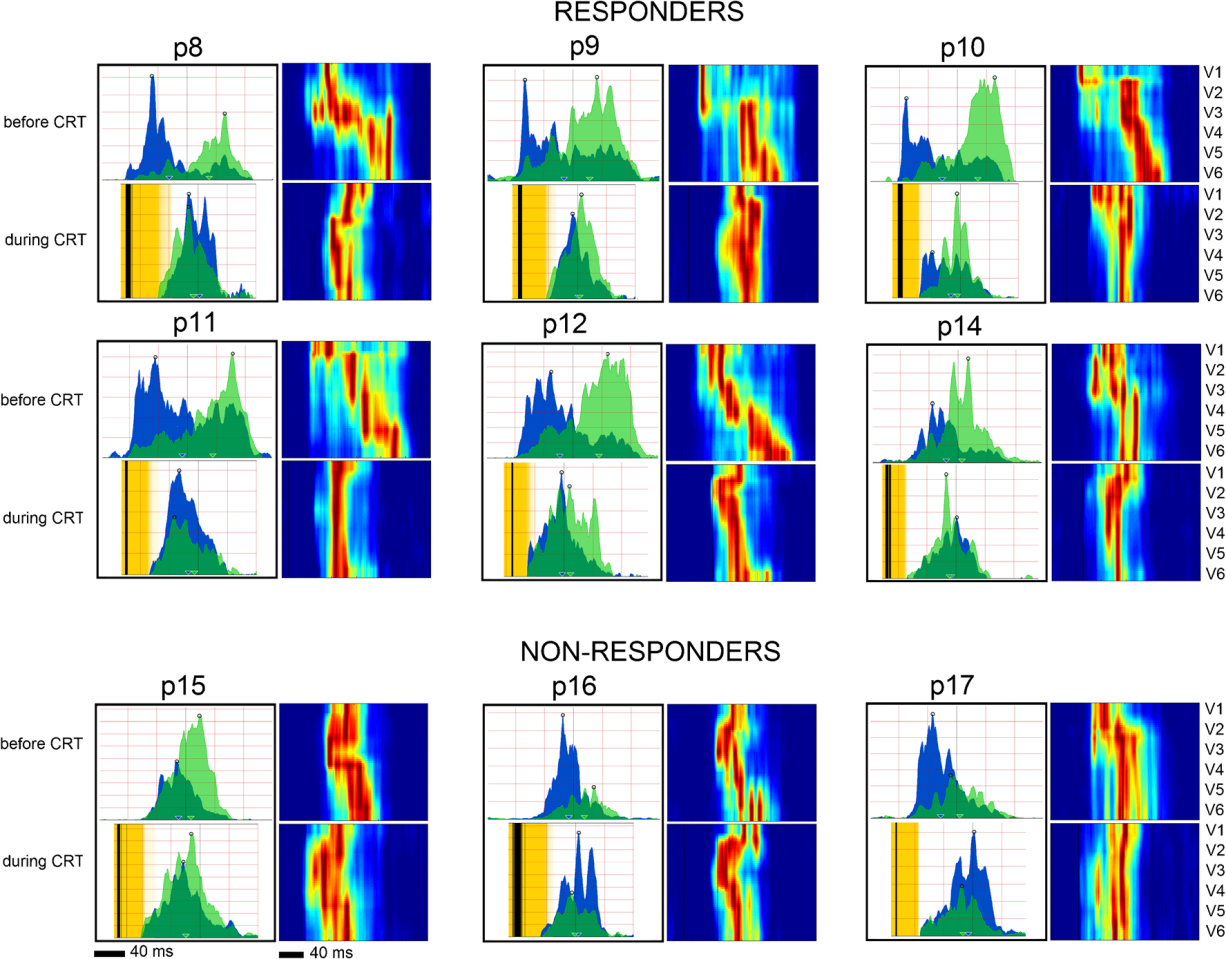
UHFQRS CRT response, 6-month follow up. Examples of nine patients with CRT. Each panel includes UHFQRS computed from V1 and V6 leads and UHFQRS maps before CRT and 6 months after CRT. All parameters are listed in Tables 1 and 2

Figure 5 introduces UHFQRS CRT responses, patients 8–12, 14 (responders), and patients 15–17 (non-responders) from Table 2. Electrical dyssynchrony DYS, QRSd, left ventricular ejection fraction (LVEF), and end-systolic volume (ESV) were measured before CRT and 6-month after CRT implantation during CRT ON. Patients 8, 9, 10, 11, and 12 represent ideal CRT recipients with significant improvement of both LV functional parameters and electrical ventricular synchrony. The wide QRS complex and large dyssynchrony before CRT are changed into a narrow QRS complex with almost no dyssynchrony - merging of blue and green V1 and V6 depolarization patterns during CRT and narrowing of UHFQRS maps. Patient 14 has the lowest DYS from the responders (53 ms). Bi-ventricular stimulation creates an alignment of electrical depolarization with slight delay in V1 lead. Patients 15, 16, and 17 shows reduced CRT effect on ventricular synchrony. There is no significant positive change in QRSd, LVEF, or ESV. UHFDYS before CRT is low. UHFQRS maps in patients 15, 16, and 17 before and during CRT are similar which means that there is very little effect of CRT on electrical synchronization.

**Fig. 6:**
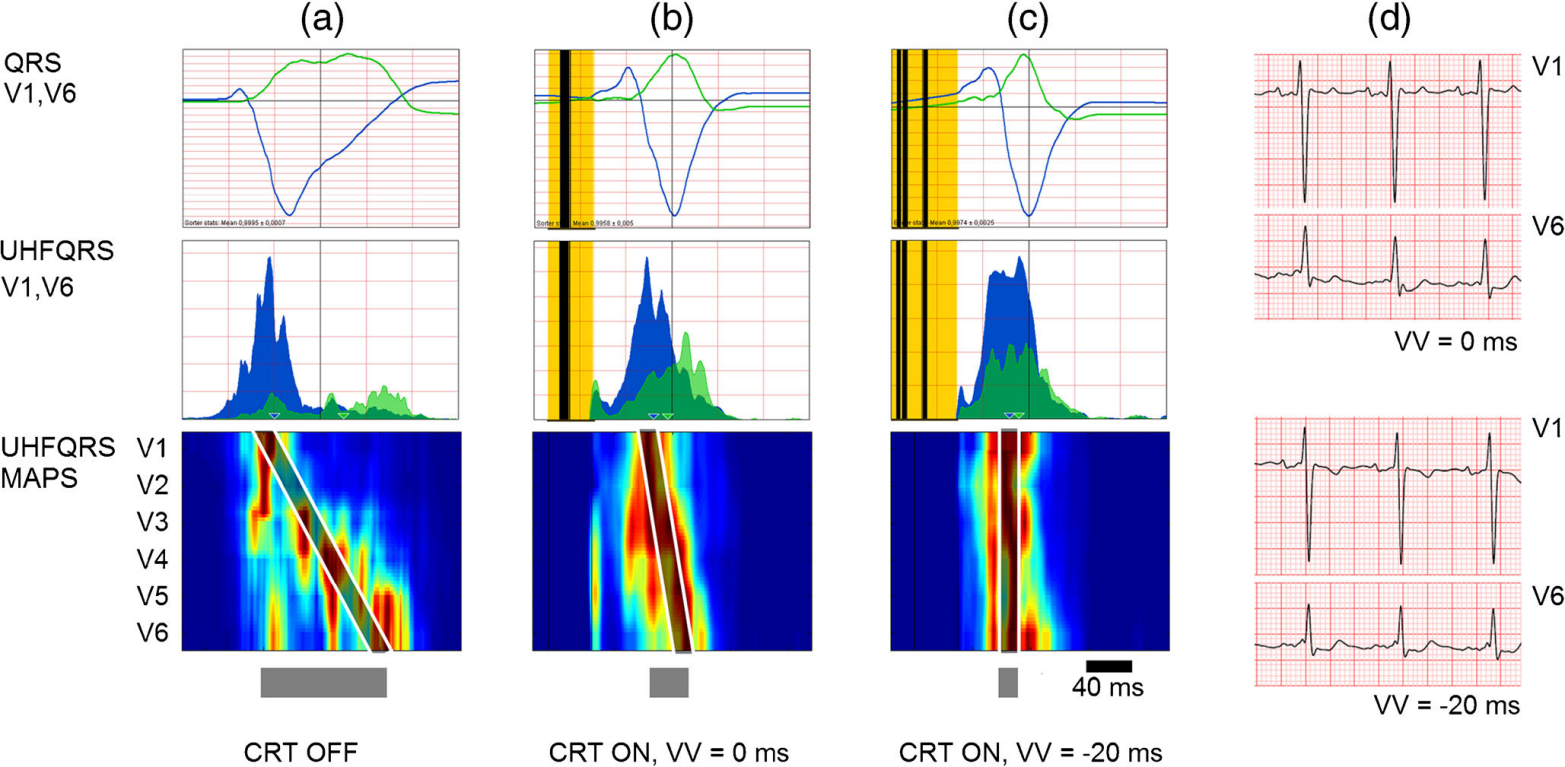
Effect of 20 ms LV lead pre-excitation on UHFDYS, patient 6. **a** CRT OFF, QRSd 194 ms, EF 25%, UHFDYS 90 ms. **b** CRT ON VV delay 0, QRSd was reduced by 62 ms, EF increased by 10%, UHFDYS 33 ms. **c** CRT ON VV delay 20 ms, QRSd remain unchanged, pre-excitation of LV lateral wall leads to further improvement of synchrony UHFDYS 23 ms. **d** V1 and V6 ECG leads, CRT ON

Figure 6 demonstrates effect of 20 ms LV lead pre-excitation on UHFQRS and UHFDYS. Patient 6 (Table 1) has large electrical dyssynchrony 90 ms and QRSd 194 ms shortened to 33 ms and 132 ms by CRT. Figure 6c depicts the positive effect of 20 ms LV lead pre-excitation. While QRSd remained unchanged (131 ms), there was significant reduction of UHFDYS (23 ms). Figure 6 demonstrates the possible role of UHFQRS method in CRT optimization when QRSd change remains less sensitive [19].

### UHFQRS maps and STE—electro-mechanical interpretation

**Fig. 7:**
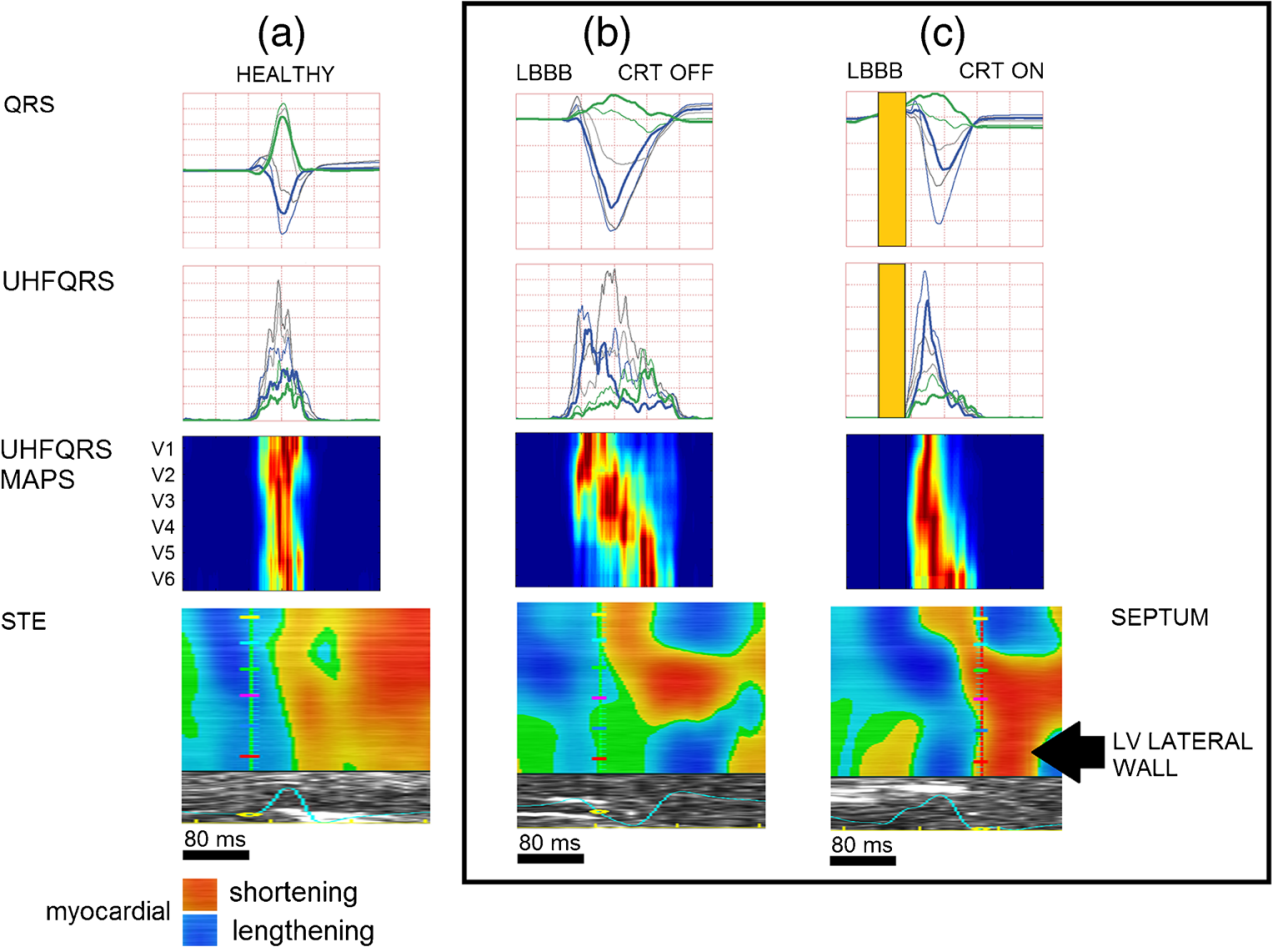
UHFQRS and STE comparison. **a** Healthy subject. **b** patient 7, CRT OFF, QRSd 193 ms, EF 25%, UHFDYS 81 ms, **c** patient 7, CRT ON, QRSd 118 ms, EF 35%, UHFDYS 6 ms, *Black arrow* indicates the effect of biventricular stimulation on earlier LV lateral wall activation in STE. The same effect can be seen in the UHFQRS map

Figure 7 shows examples of UHFQRS maps and STE maps. In a healthy heart (Fig. 7a), mechanical deformation begins synchronously in all parts of the LV, confirmed by the narrow QRS, overlapping UHFQRS over V leads, and the single line in the UHFQRS maps corresponding to the orange/red edge of STE.

Figure 7b, c show the effect of biventricular pacing (patient 7). The black arrow localizes the positive shift of the onset of deformation of the LV lateral wall and the significant reduction in mechanical dyssynchrony in the STE. The same positive effect can be seen in UHFQRS maps. While STE examination requires good quality echocardiographic imaging and cannot be performed on every patient, UHFQRS does not face the same restrictions.

## Discussion

The gold standard for detailed assessment of RV or LV dyssynchrony is still being discussed [1]. Surface ECG represents the oldest and most widely used method for diagnosing pathological electrical activation of the myocardium by comparing the shape and duration of the QRS complex in a 12-lead ECG. Large randomized studies claim that the best criteria for CRT candidate selection are a QRS duration of >150 ms and true LBBB morphology of the QRS complex [20]. CRT guidelines limit QRS duration to 120 ms [21]. Strauss criteria that include a QRS duration ≥140 ms for men and ≥130 ms for women, along with mid-QRS notching or slurring in ≥2 contiguous leads [1], currently represent the most powerful marker for complete LBBB identification. However, ECG represents a global view of electrical activity of the heart with no direct information about mechanical activity.

We compared ten patients (Table 2) before CRT and 6-month after CRT during CRT ON. We present ECG parameters (QRSd, QRS morphology—Strauss criteria and UHFQRS dyssynchrony) and echocardiographic LV functional parameters (LVEF, ESV). Patients 8–14 show a positive effect of CRT on LV function. The QRS morphology before CRT meets the Strauss criteria (patients 8, 9,11,12, and 13), and UHFQRS before CRT shows a large electrical depolarization delay between the septum and LV lateral wall. Patient 10 does not meet Strauss criteria (no notch or slur), but has wide QRSd and high UHFDYS. Patient 14 has the lowest level of DYS, the shortest QRS of all responders, and does not meet Strauss criteria. Even so, there is clear synchronization effect (Fig. 5, p14).

Patients 15–17, classified as non-responders, have small UHFQRS dyssynchrony (<50 ms) and narrow QRSd (<151 ms) prior to CRT. Biventricular pacing has no effect on QRSd and LV ESV. CRT reduces electrical dyssynchrony, though significantly less than in responders.

Our preliminary results of ten consecutive patients who completed 6-month follow-up suggest the potential of UHF-ECG derived dyssynchrony to correctly define responders or non-responders to CRT. Thus, UHF-ECG derived dyssynchrony deserves attention and, considering also advantages of UHF-ECG mentioned in the text of our paper, it may have the potential to be one of the most significant predictors of responsiveness to CRT.

Mechanical cardiac activity can be assessed primarily by echocardiography or magnetic resonance imaging (MRI). Several echocardiographic methods have been clinically utilized for the quantification of mechanical (contractile) myocardial dyssynchrony, including M-mode echocardiography, Doppler tissue imaging, Doppler-derived strain, and strain rate analysis or, more recently, STE. Each of these methods has limitations leading to modest accuracy to predict CRT response [22, 23]. Lower reproducibility and temporal resolution over 10 ms are the main drawbacks. MRI is a rapidly developing non-invasive technique for monitoring cardiac mechanics. It provides valuable spatial imaging of cardiac tissues and their dynamic change. The main limitation of MRI is its availability for routine diagnostics and its cost.

High UHFQRS temporal resolution, resulting from the frequency band, is coupled with superior reproducibility. Sufficient signal-to-noise ratio is obtained by averaging. The averaging technique cannot be used in echocardiography. Moreover, the analysis may be conducted with various types of QRS complexes, and even irregular beats can be independently analyzed.

If the premise that UHFQRS measures the activity of depolarizing action potentials of myocardial contractile cells (Phase 0 of AP) is accepted, the electrical and mechanical activity of the ventricles can also be put into context (Figs. 3 and 7). Leclerq et al. [24] showed in a canine study that the course of mechanical contraction (during LBBB, LV paced only and biventricular paced states) was different from the electrical depolarization of ventricles. However, the initial phase of mechanical contraction corresponded with myocardial depolarization. Kroon et al. [25] introduced a comparison of LV electrical activation and mechanical shortening delays in ten heart failure patients with prolonged QRSd. The conclusion is that there are intra-individual and inter-individual differences in depolarization time and mechanical shortening, though overall depolarization maps strongly correlate with time-to-peak mechanical maps. This is in agreement with our findings about the connection between the ventricular electrical depolarization and the initial phase of mechanical activation. The assumption of a relationship between electrical depolarization and mechanical activation makes the analysis of electrical depolarization dyssynchrony by UHFQRS an important instrument.

### Limitations

Here, we present a new technique based on limited examples. We are aware that these preliminary results serve rather as a demonstration of the potential of the UHFQRS method.

UHF-ECG measures and evaluates very weak voltage potentials far below the level of microvolts. The lower signal-to-noise ratio must be eliminated by prolongation of the measurement time to obtain more QRS patterns to average (usually 3–5 min).

Technical limitations: We demonstrate UHFQRS in a frequency range of 500–1000 Hz. To validate the presented results, ECG monitors with internal sampling higher than 2 kHz and minimal 16-bit or higher analog-digital converters are needed. Conventional 1 kHz ECG and frequency range 150–350 Hz seems to be sufficient to obtain basic information about ventricular dyssynchrony.

### Positives

UHFQRS is a deterministic method. In this proof-of-concept study, the clinical operator had no access to the UHFQRS maps and UHFQRS morphology computation.

A significant advantage of the UHFQRS method is simplicity in application: Standard 12-lead ECG electrode placements can be used. The UHFQRS amplitude shape and multi-lead amplitude overlap can be identified and parametrized, unlike the morphology of the QRS complex which is not always clear (Fig. 1d, e). Moreover, irregular beats can be analyzed and compared with sinus beats. Such analysis is nearly impossible with echocardiographic methods.

## Conclusion

The purpose of this study was to demonstrate the technical feasibility and the potential ability to assess the temporal-spatial distribution of LV depolarization. UHFQRS is an easily accessible and simple technique with the potential to provide valuable information about myocardial electrical dyssynchrony that could be related to the initial point of mechanical activation. The major clinical potential of UHFQRS may lie in more accurate identification and selection of CRT recipients and optimization of biventricular pacing parameters. Further retrospective and prospective studies with high resolution ECGs will be needed to define the appropriate role of this new technology.

## Electronic supplementary material

ESM 1 (DOC 5794 kb)
