# Supplementary material for: Ventricular dyssynchrony assessment using ultra-high frequency ECG technique
Source: J Interv Card Electrophysiol. 2017 Jul 10;49(3):245–54. doi: 10.1007/s10840-017-0268-0 (PMC5543201; doi:10.1007/s10840-017-0268-0)
Supplement: Supplementary file 1 — (DOC 5794 kb) [file 10840_2017_268_MOESM1_ESM.doc]

**Supplementary Material**

Article title: **Ventricular Dyssynchrony Assessment using Ultra-High Frequency ECG Technique**

Journal: **JICE**

Author names:

Pavel Jurak^1^ PhD,

Josef Halamek^1,2^ PhD,

Jaroslav Meluzin^2,3^ MD, PhD,

Filip Plesinger^1^ PhD,

Tereza Postranecka^2^ MSc,

Jolana Lipoldova^2,3^ MD, PhD,

Miroslav Novak^3^ MD, PhD,

Vlastimil Vondra^1,2^ PhD,

Ivo Viscor^1^ PhD,

Ladislav Soukup^2^ MSc,

Petr Klimes ^1,2^ MSc,

Petr Vesely^2^ MSc,

Josef Sumbera^2,3^ MD, PhD,

Karel Zeman^2,3^ MD, PhD,

Roshini S. Asirvatham^4^,

Jason Tri^4^,

Samuel J. Asirvatham^5,6^ MD

Pavel Leinveber^2^ MSc

List of the departments and institutions:

^1^ Institute of Scientific Instruments of the Czech Academy of Sciences, Czech Republic

^2^ International Clinical Research Center, St. Anne’s University Hospital, Brno, Czech Republic

^3^ 1^st^ Department of Internal Medicine-Cardio-angiology, St. Anne’s University Hospital, Masaryk University, Brno, Czech Republic,

^4^Student Scholar Program, Mayo Clinic, Rochester;

^5^Division of Cardiovascular Diseases, Department of Internal Medicine, Mayo Clinic, Rochester, Minnesota,

^6^Department of Pediatrics and Adolescent Medicine, Mayo Clinic, Rochester, Minnesota

Address for correspondence:

Pavel Jurák, Ph.D.

Institute of Scientific Instruments of the CAS, v.v.i.

The Czech Academy of Sciences

Kralovopolska 147, 61264, Brno

Czech Republic

Phone: +420 608 821 356

Fax: +420 541 514 402

E-mail: jurak@isibrno.cz

Here we describe the Ultra-High Frequency ECG (UHF-ECG) data analysis methods in more detail and with additional examples.

Custom-made UHF Solver software (ISI CAS, Brno, CZ) specifically developed for UHF-ECG data processing was used. This software performs the elimination of stimulating peaks, R-wave detection, QRS complex sorting, amplitude envelope computation, averaging, smoothing, visualization and numerical descriptor detection.

**Biventricular pacing ON – removal of stimulation peaks**

Stimulation peaks generate high-frequency components and negatively affect the UHF-ECG oscillations. Special efforts were devoted to defining the area between the end of the stimulation peak region and the beginning of the QRS complex. If the peak interfered with the QRS complex, it was not possible to analyze the UHF components.


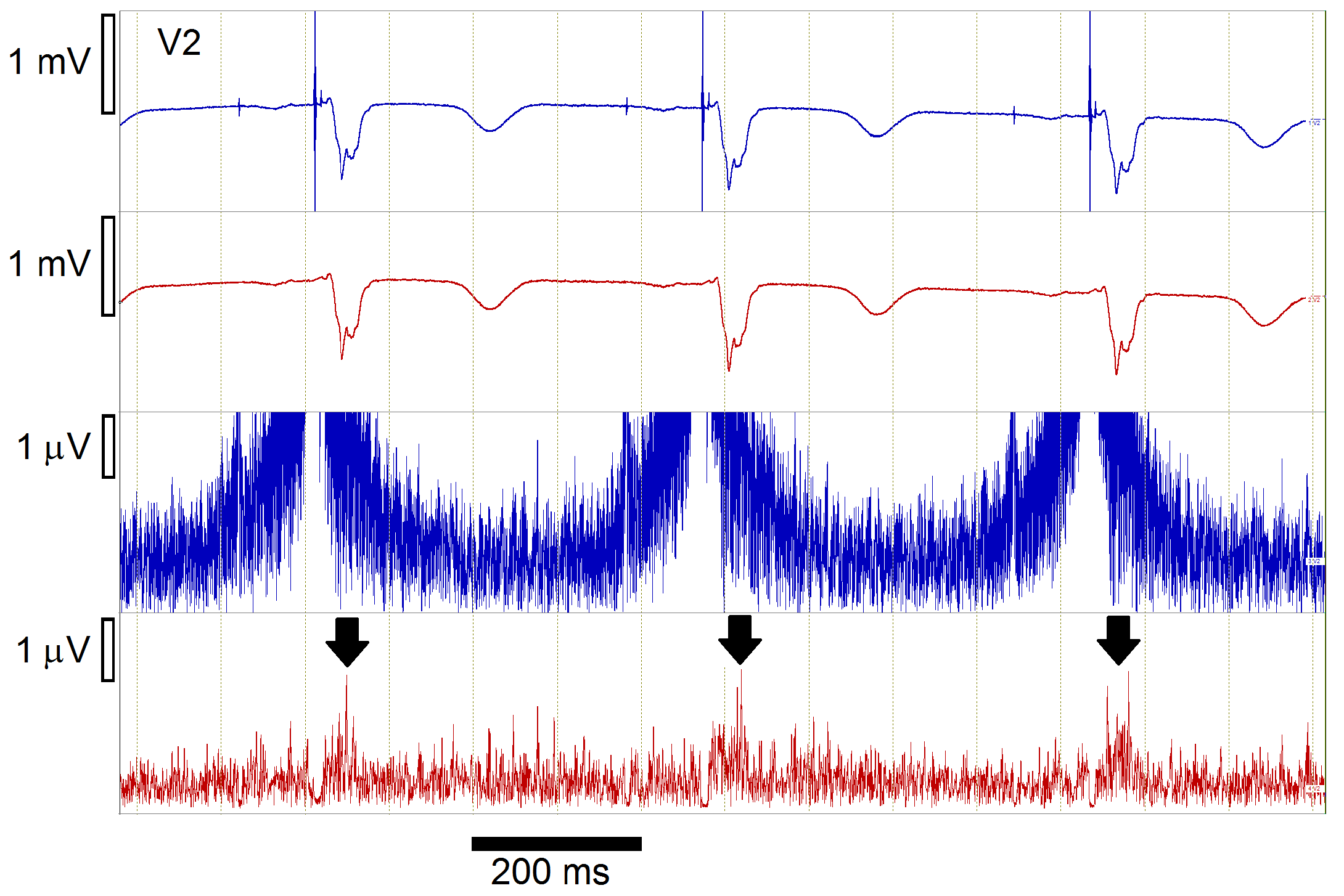


*Figure S1:Stimulation UHF artefacts.*

*From the top: three QRS complexes (V2 lead) with stimulation artefacts (blue color), QRS complexes after elimination of stimulation artefact (red color), amplitude envelopes 500-1000 Hz with artefacts (blue) and with artefacts removed (red). Black arrows mark an increase in UHF oscillations in the QRS complex region.*


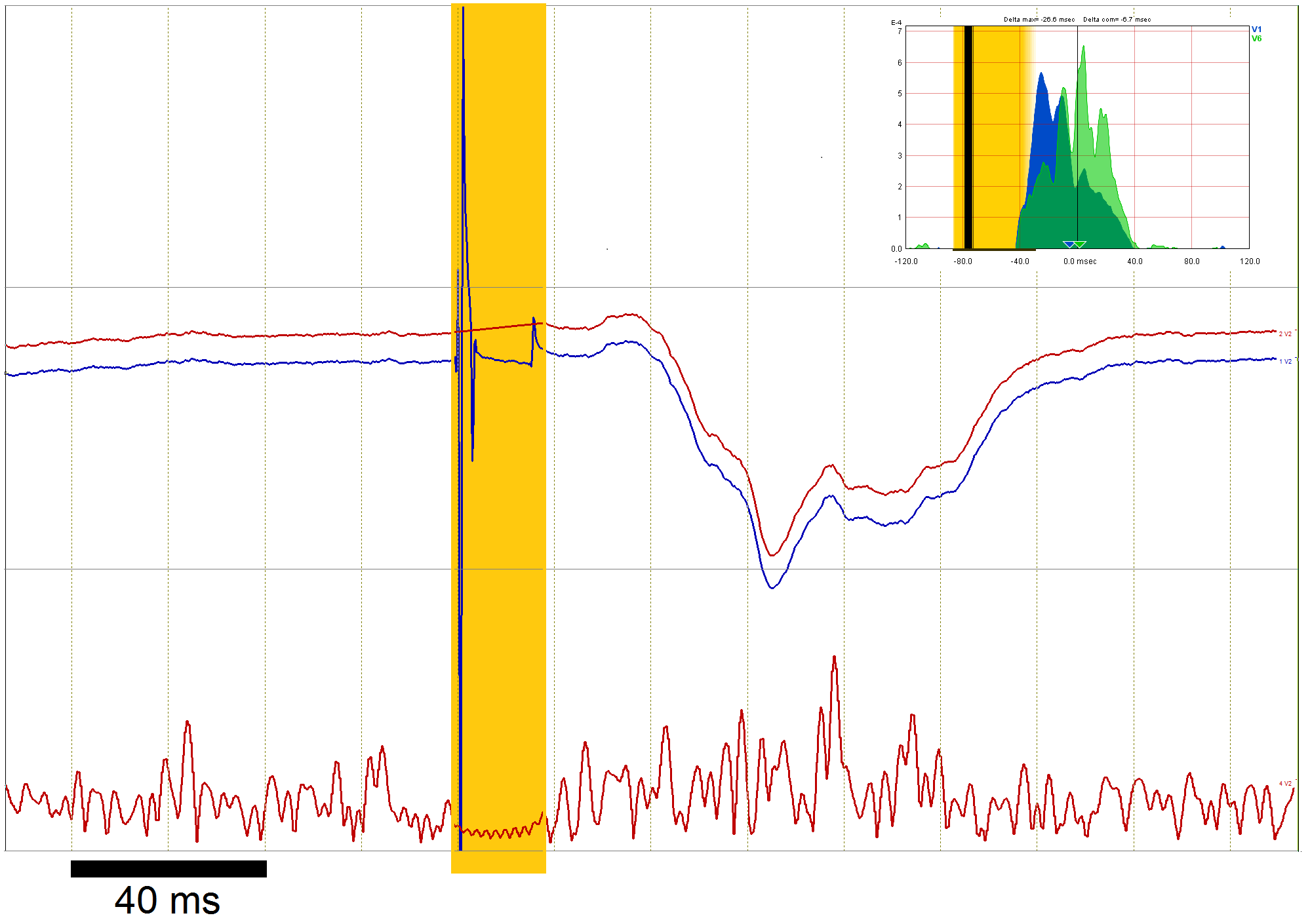


*Figure S2: Detail of V2 lead QRS complex with/without stimulation artefact (top) and amplitude envelopes 500-1000 Hz (bottom).*

*The yellow rectangle defines the area affected by stimulation artefacts elimination that cannot be used for UHF component analysis.* *The result of automatic processing by the program UHF Solver is shown in the top right corner – V1 and V6 lead averaged and smoothed amplitude envelopes. In this case, the stimulus sufficiently precedes the QRS complex and does not affect the UHF components. If the pacemaker stimuli are positioned in the QRS region, the UHF components can be analyzed only in a limited part of the QRS.*

**R-wave detection and categorization (sorting)**

QRS complexes of V leads are detected and sorted using a robust multichannel correlation algorithm (15, Plesinger, Computing in Cardiology, 2014). Sorting is crucial for differentiation of QRS morphologies and irregularities. Figure S3 represents an example of QRS categorization into two groups. This classification is performed according to the value of the correlation coefficient. This technique is used to differentiate any artificial or abnormal QRS shapes.

*
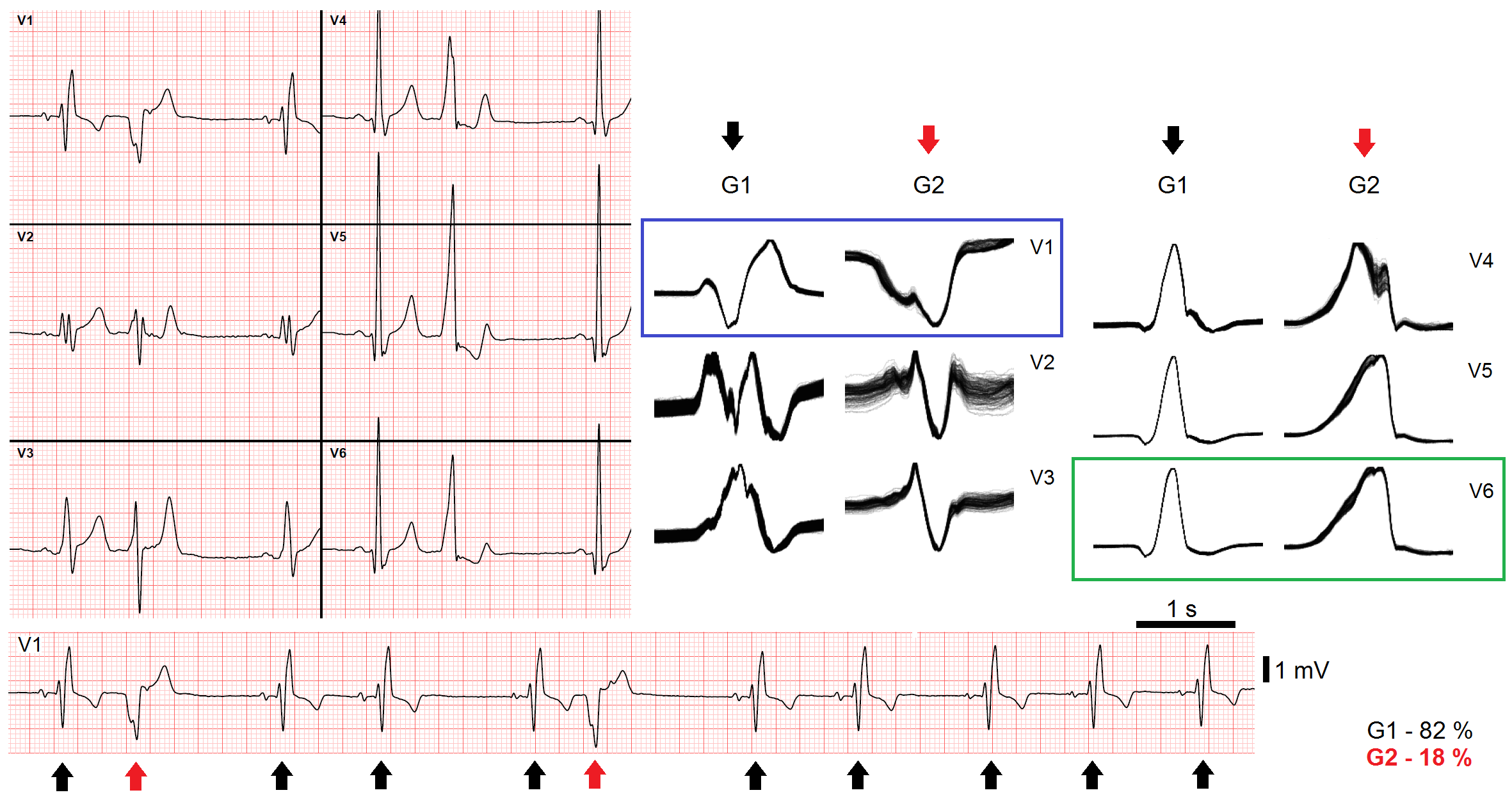
*

*Figure S3A: UHF-ECG – QRS complex detection and categorization.*

*Left panel: the V1-V6 leads in standard clinical form. Right panel: two QRS complex categories – group G1 (sinus rhythm, 82 %) and G2 (premature ventricular contraction, 18 %).*

*
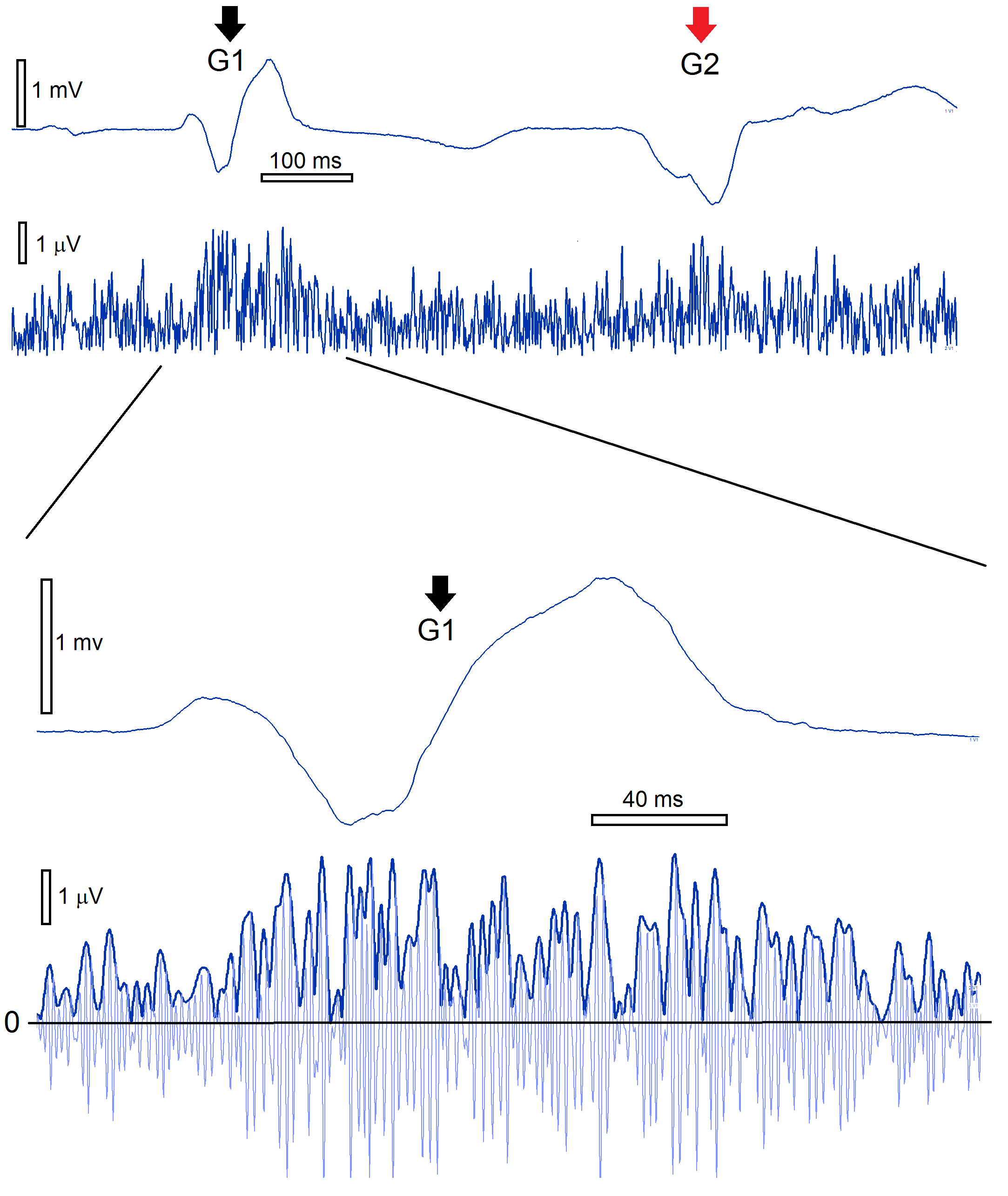
*

*Figure S3B: UHF-ECG – QRS complex filtration and envelope computation. Upper panel: two consecutive heart beats in V1 lead, group G1 and group G2 QRS complexes and UHF amplitude envelope 500-1000 Hz (dark blue). Bottom panel – zoom of G1 QRS complex in V1 lead and filtered UHF-ECG signal in passband 500-1000 Hz (light blue) and UHF amplitude envelope 500-1000 Hz (dark blue). The UHF amplitude is more than a thousand times smaller than the standard QRS amplitude (mV versus micro V).*

*
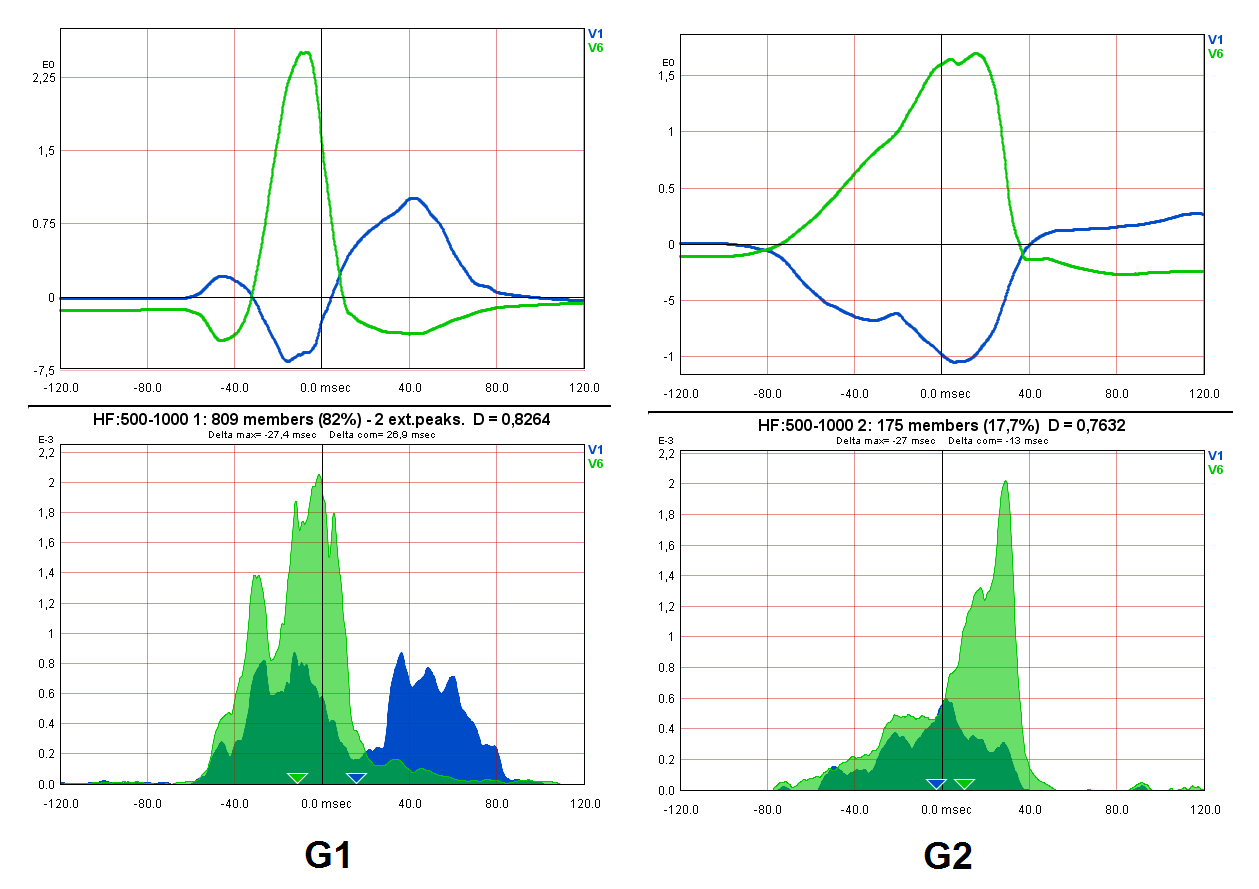
*

*Figure S3C: Averaged QRS and UHF components, G1 and G2 groups.*

*Upper panels: averaged QRS, lead V1 (blue) and V6 (green). Bottom panels: averaged and smoothed UHF 500-1000 Hz amplitude envelopes (UHFQRS), separately group G1 (left) and G2 (right). Total number of G1 QRS complexes is 809 (82 %) and G2 QRS is 177 (18 %) in 15 minutes record. Averaging from 809 beats in G1 improves signal-to-noise ratio by a factor of around 25, averaging from 117 beats in G2 improves signal-to-noise ratio by a factor of around 10. This is sufficient to determine the UHF properties in selected leads.*

**Amplitude envelopes computation, averaging and smoothing, normalized maps**

The amplitude envelopes in passband 500-1000 Hz were computed using the Hilbert transform (Figure S3B, bottom panel), averaged with an R-wave trigger and smoothed in the passband 0-40 Hz (UHFQRS).


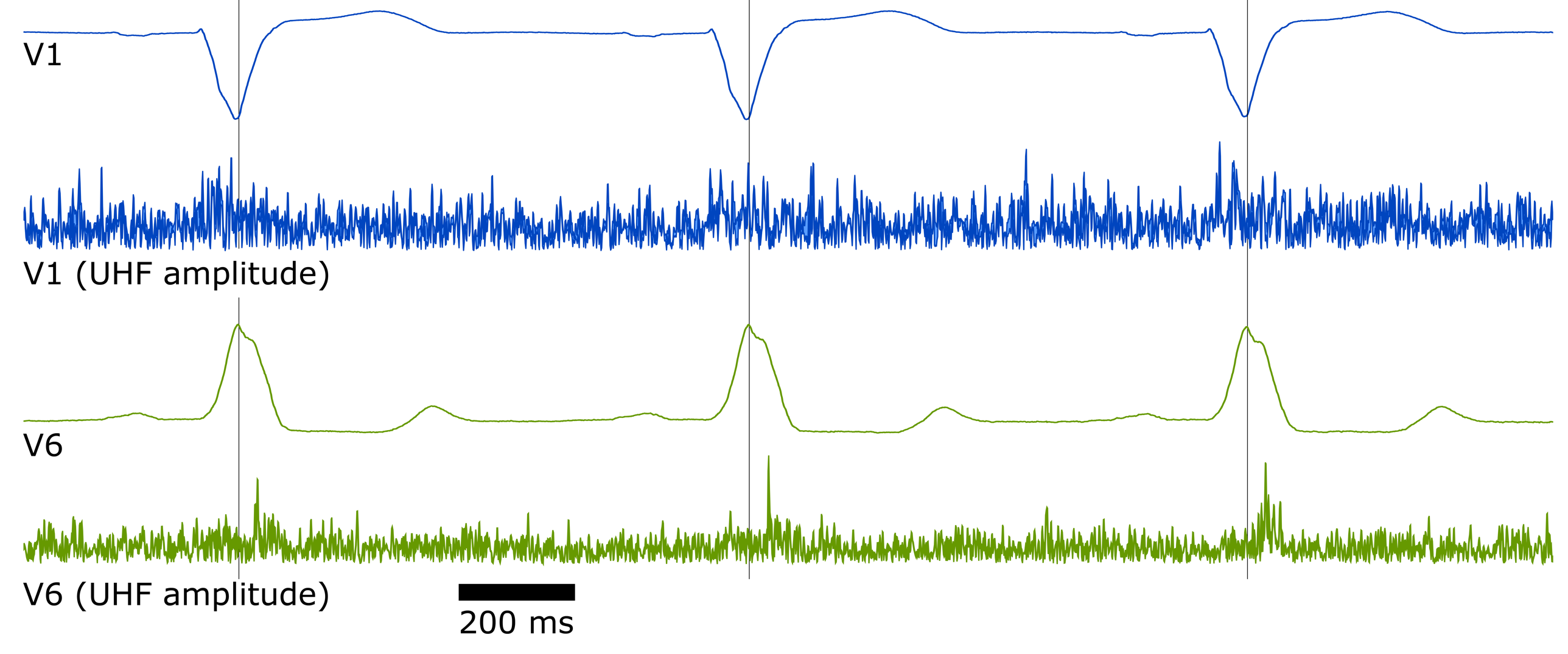


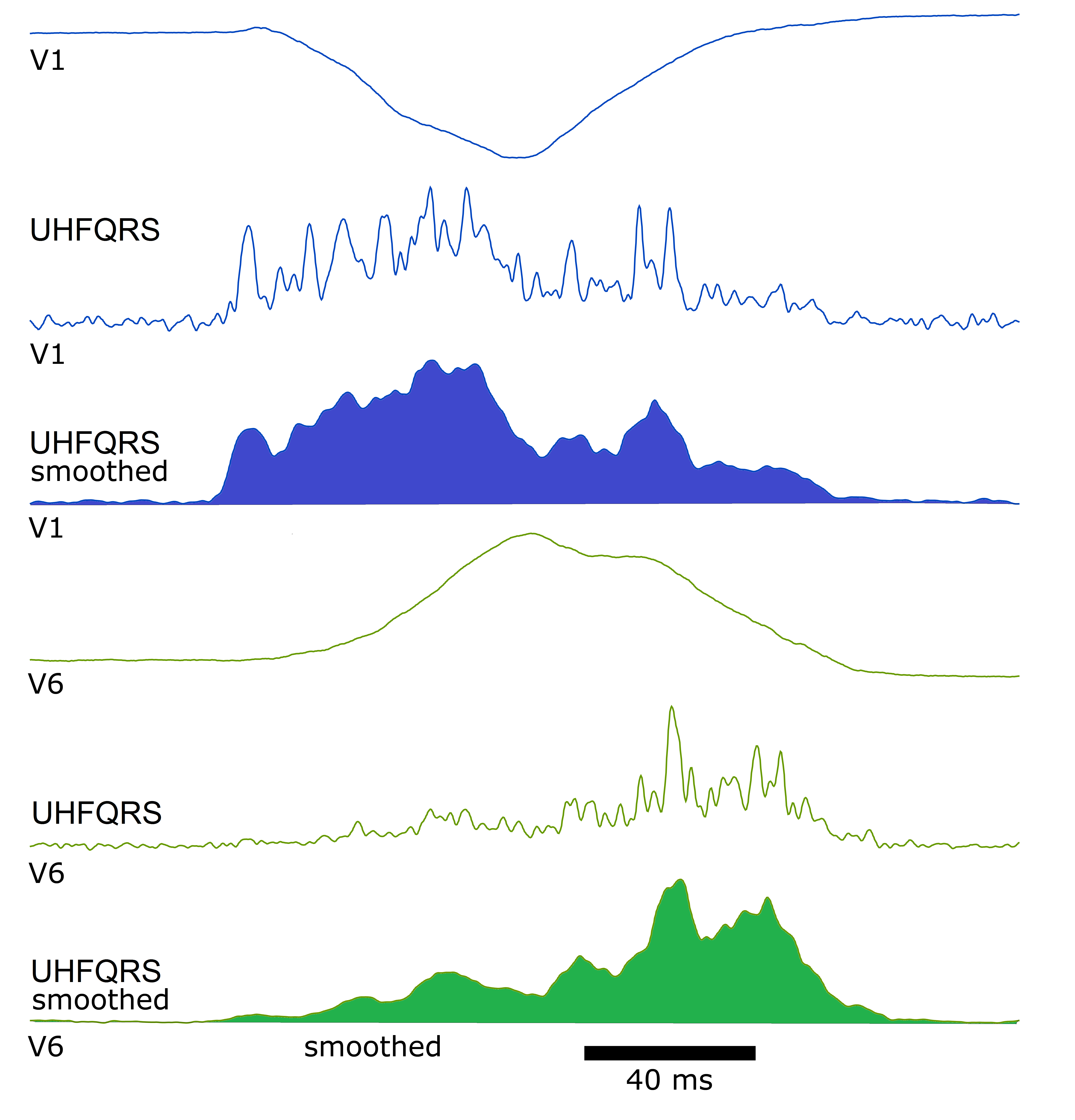

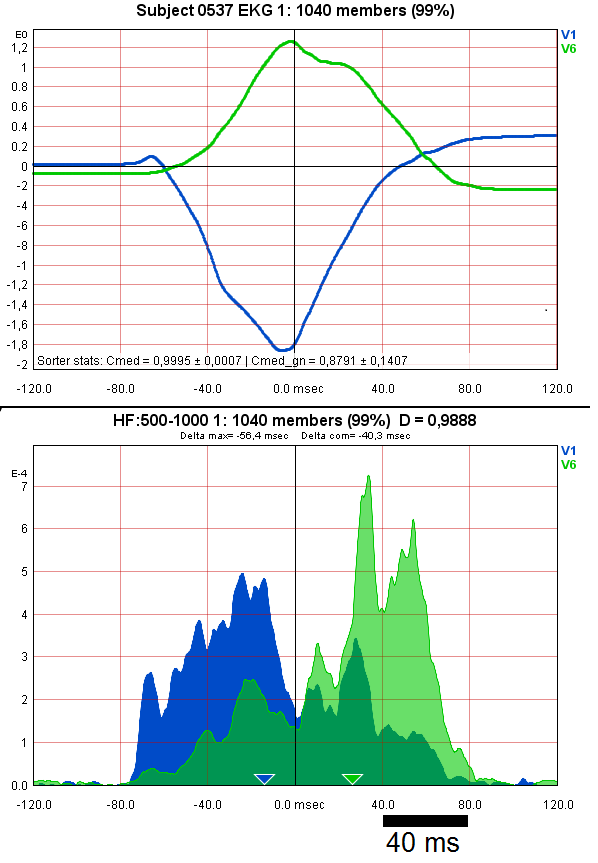


*Figure S4: Consecutive steps of Ultra-High Frequency Averaged QRS Amplitude Envelopes (UHFQRS) computation and visualization.*

*Upper panel from top: raw V1 (green) and V6 (blue) ECG signals (5 kHz sampling) and amplitude envelopes (500-1000 Hz).*

*Bottom panel left: averaged QRS complexes V1 and V6 (1040 beats), averaged amplitude envelopes non-smoothed UHFQRS and smoothed UHFQRS in frequency range 0-40 Hz.*

*Bottom panel right: resulting shape of smoothed V1 and V6 UHFQRS in overlapping color representation.*

*
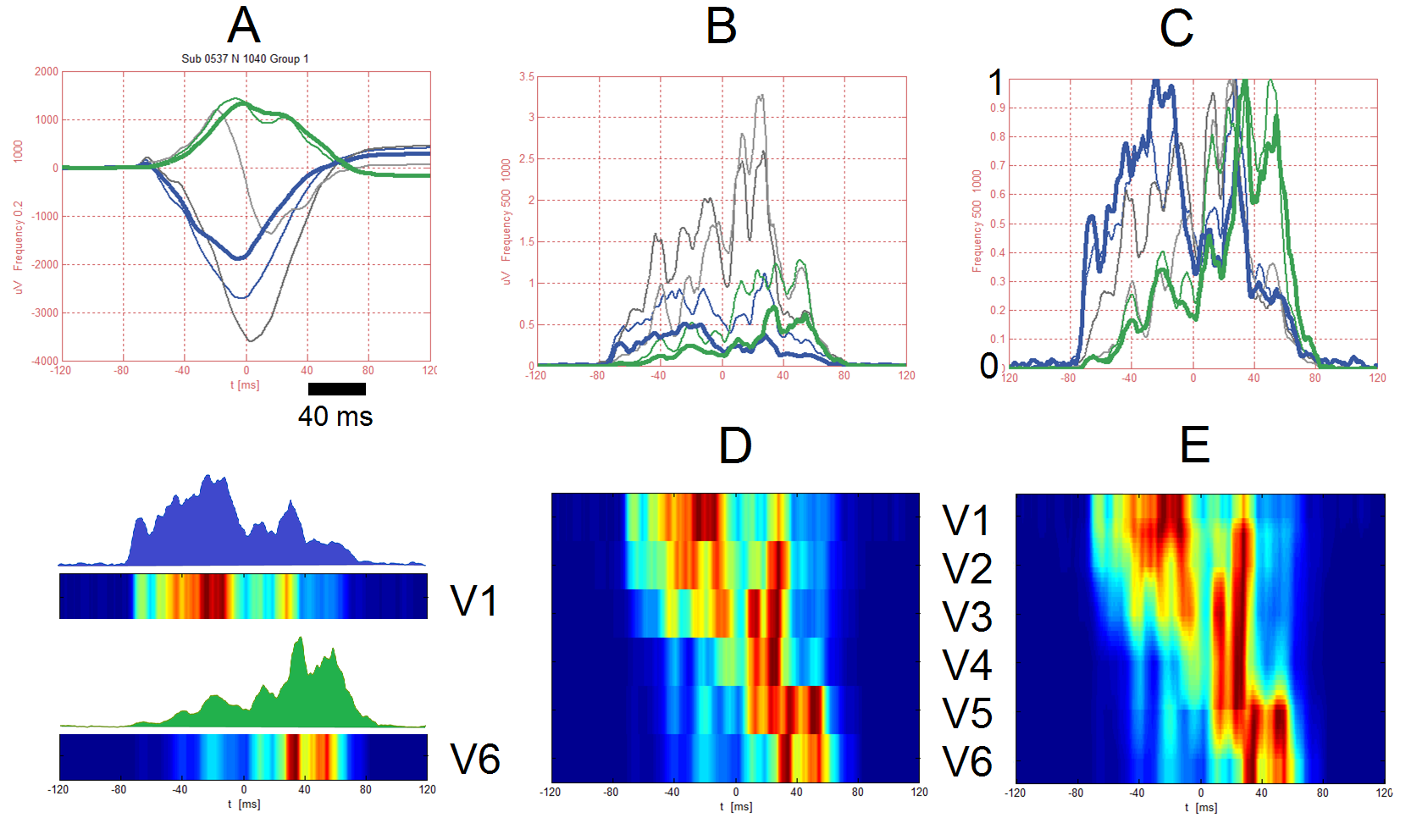
*

*Figure S5: Normalized UHFQRS map compilation.*

*A: averaged QRS complexes V1-V6, V1 – thick blue line, V2 – thin blue line, V3 – dark gray line, V4 – light gray line, V5 – thin green line, V6 – thick green line.*

*B: UHFQRS V1-V6.*

*C: normalized UHFQRS. The maximum in each lead is normalized to 1 and the minimum is normalized to 0.*

*D: UHFQRS map – the horizontal row of the map (V1, V2, … ,V6) represents the normalized shape of UHFQRS for one V lead, dark red means 1, dark blue means 0.*

*E: smoothed map – linear interpolation is used between rows (leads).*

**Frequency bands – HF and UHF**

The UHFQRS in the frequency range 500-1000 Hz are presented in the manuscript. The envelopes in the HF 150-250 Hz and UHF 1000-2000 Hz frequency bands were simultaneously computed. HF frequency band ECG has been previously reported for the diagnosis of ischemia (HFQRS, Amit, 2014) and 150-250 Hz represents the persistent and generally accepted range of broadband HF ECG. The frequency band UHF 1000-2000 Hz represents the upper limit of UHF and is more sensitive to external interference and noise. Improving signal to noise ratio requires a prolongation of UHF-ECG recordings in noisy environments. The frequency band 500-1000 Hz would, therefore, seem to be the optimal compromise between measurement length, signal-to-noise ratio and spatial-temporal resolution.

The samples in Figure S6 demonstrate averaged QRS complexes and amplitude envelopes in HF and two UHF frequency ranges. The shapes of standard V1-V6 leads (QRS complex) are accompanied by the HF and UHF amplitudes in a healthy heart (A), a heart with right bundle branch block (RBBB) (B) and a heart with left bundle branch block (LBBB) (C). The approximate identification of dyssynchrony is possible from the HF band. However, the time position of the depolarization peak cannot be precisely detected without wide smoothing and, therefore, a reduction of the temporal resolution in the HF band. UHF peak position is crucial for precise estimation of dyssynchrony in milliseconds. The temporal resolution increases and the signal-to-noise ratio decreases with increasing carrier frequency and, in particular, frequency band width. The frequency band width 500-1000 Hz appears to be an optimal compromise, especially when measurements are shorter than 5 minutes.

Comment: the manuscript primarily discusses ventricular dyssynchrony. It does not consider the morphology of single-lead depolarization distribution. This morphology reflects electrical and structural inhomogeneity in the ventricular region delimited by the lead. The higher frequency and, in particular, the higher frequency band width allows for much more precise analysis of local structural changes.

**
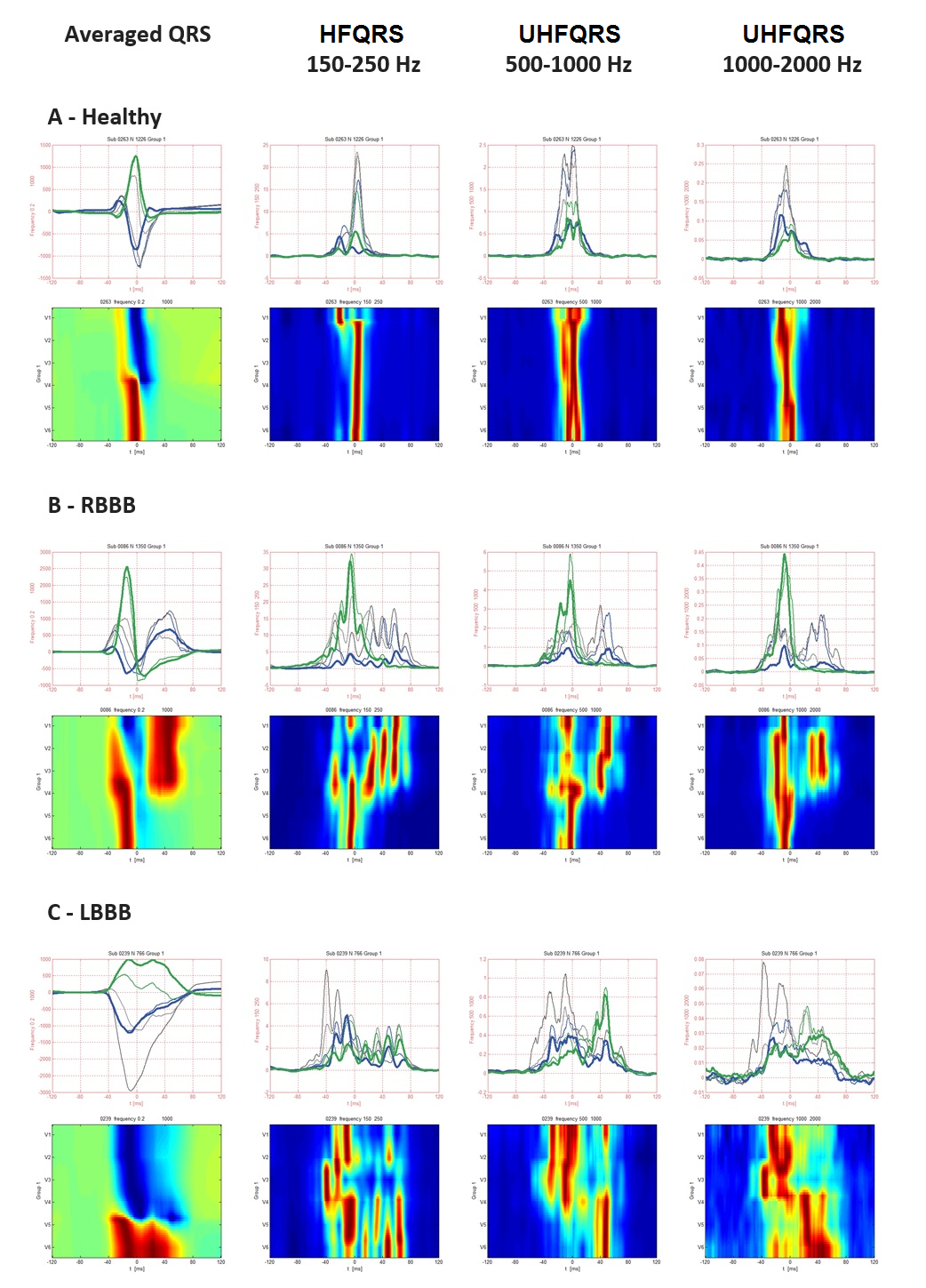
**

*Figure S6: Averaged amplitude envelopes of HF (150-250 Hz, HFQRS) and UHF (500-1000, 1000-2000 Hz, UHFQRS) on dominant group of QRS complex through V1 – V6 leads.*

*Panel A: healthy heart, panel B: RBBB patient, panel C: LBBB patient.*

*Top row in each panel: averaged QRS complex, averaged amplitude envelopes 150-250 Hz, 500-1000 and 1000-2000 Hz, blue color – V1 (thick line) and V2, green color – V5 (thick line) and V6. Bottom row in each panel: normalized HFQRS and UHFQRS maps over V1-V6 leads.*

*The delayed UHFQRS in the V1, V2 and V3 leads is presented in a selected RBBB patient; conversely, in an LBBB patient, the V5 and V6 leads shows late activity. The delay of late depolarization activation can be localized and precisely determined in UHFQRS. HFQRS does not provide this information with such temporal accuracy.*

**The individual contribution of ECG leads to UHF dyssynchrony**

Ventricular dyssynchrony is predominantly diagnosed from 12-lead ECG measurement and QRS complex morphology in I, aVR, V1 and V6 leads (4, Vernooy, Nat Rev Cardiol 2014). Figure S7 A, B, C compares the UHF oscillation between the 12 leads in an LBBB patient. The rectangles in Figure S7A indicate features that meet various guideline criteria – QRS duration (162 ms), QRS morphology in I, aVR, V1 and V6 leads. Figure S7B shows averaged QRS complexes and UHFQRS of I, aVR (left) and V1, V6 (right) leads. In the I and aVR leads, the UHFQRS overlaps and the dyssynchrony cannot be identified; in the V1 and V6 leads, the UHFQRS are split and clearly define a 78 ms delay. Figure S7C compares QRS and UHFQRS normalized maps of all 12 leads. While the different QRS shape can be seen in all leads (C1), the UHFQRS change can be seen only in V leads (C2). The UHFQRS computed on I, II, III, aVL, aVR and aVF leads does not provide information about ventricular dyssynchrony distribution. The reduction on V1-V6 leads considerably simplifies dyssynchrony identification and interpretation without loss of information.

*
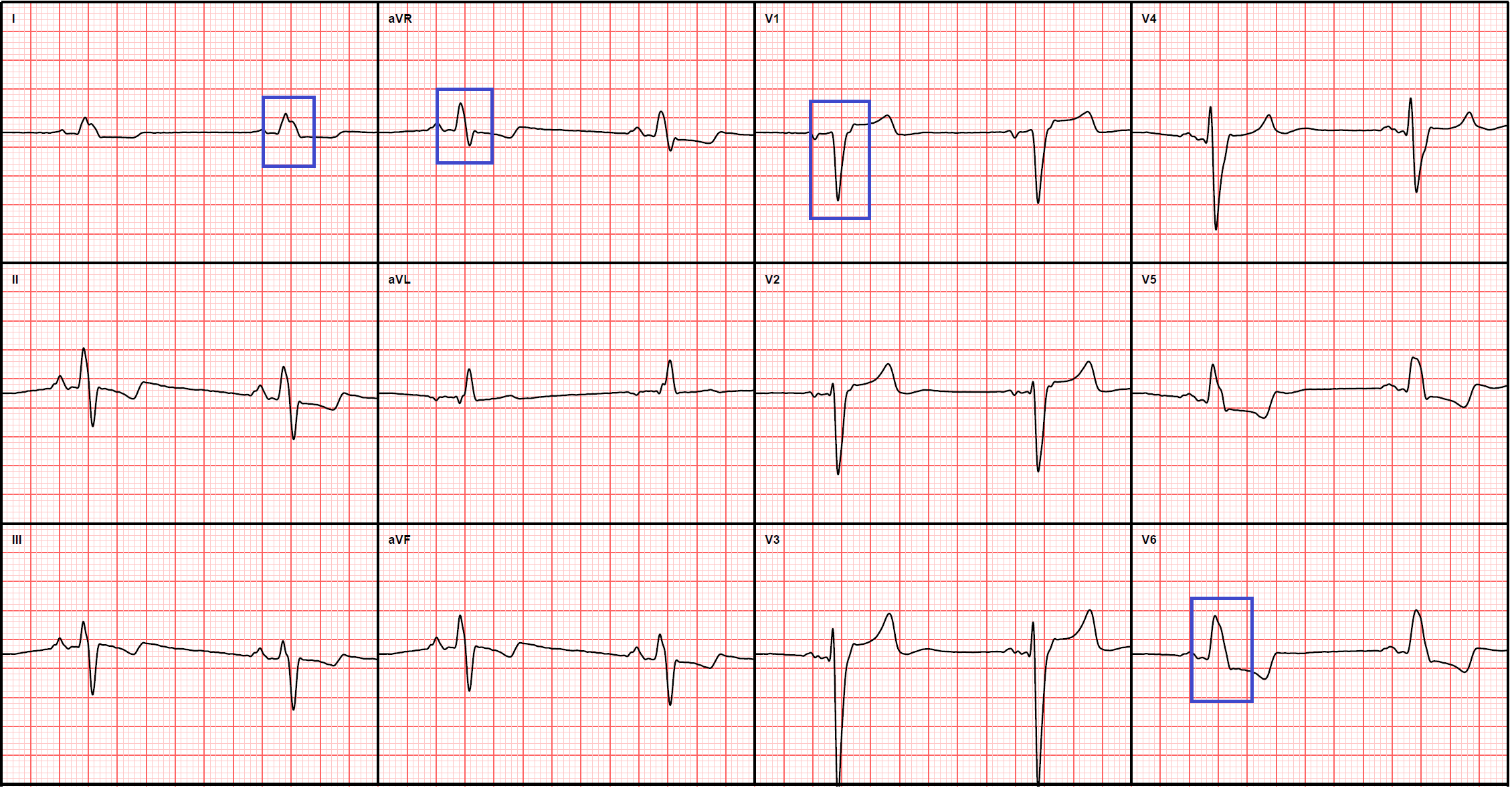
*

*Figure S7A: The electrocardiogram - typical LBBB morphology.*

*The rectangles indicate features that meet various guideline criteria – QRS duration 162 ms, LBBB specific QRS morphology in I, aVR, V1 and V6 leads.*

*
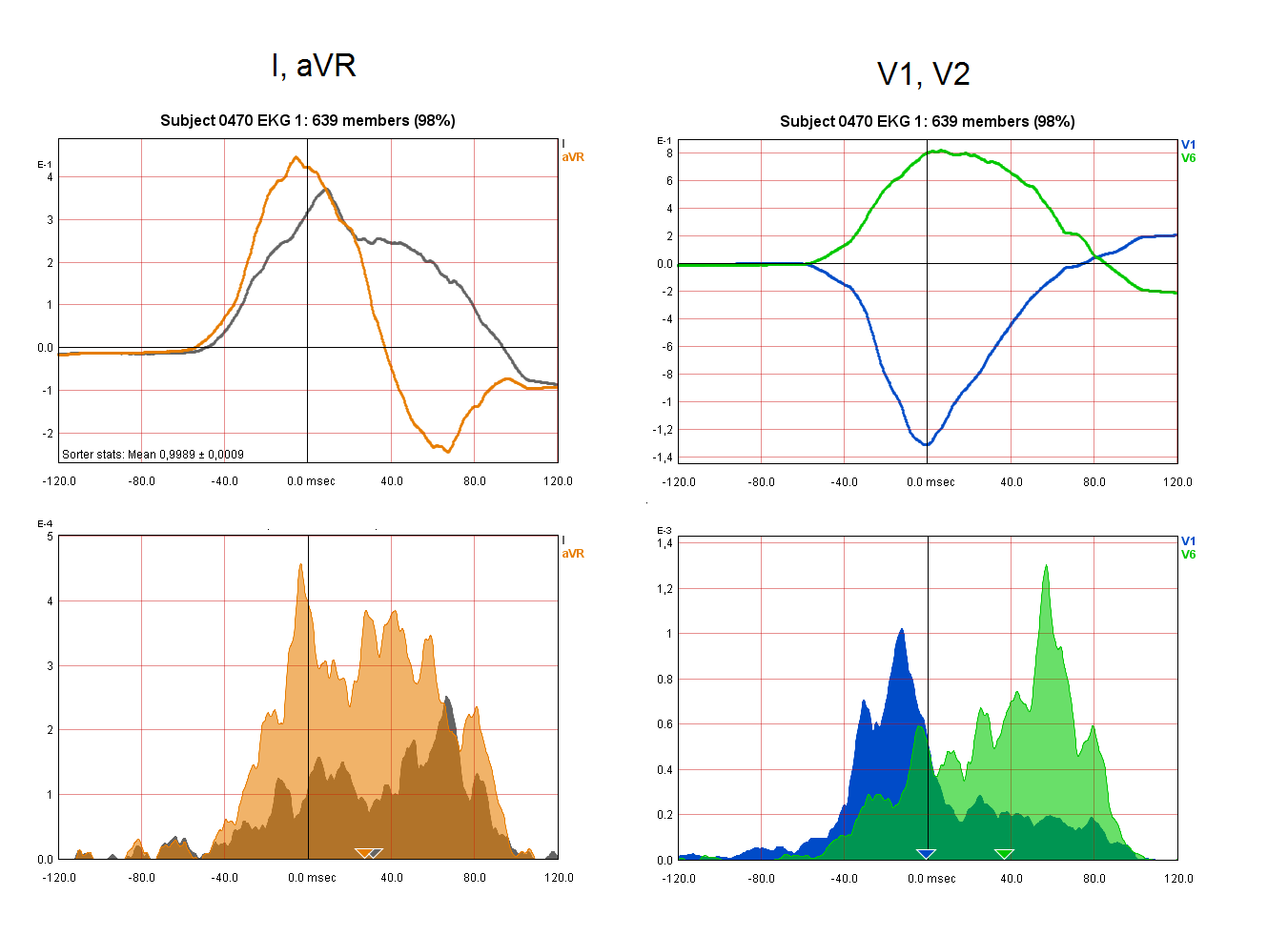
*

*Figure S7B:* *Averaged QRS complexes and UHFQRS.*

*I, aVR (left, brown and orange) and V1, V6 (right, green and blue) leads.*

**
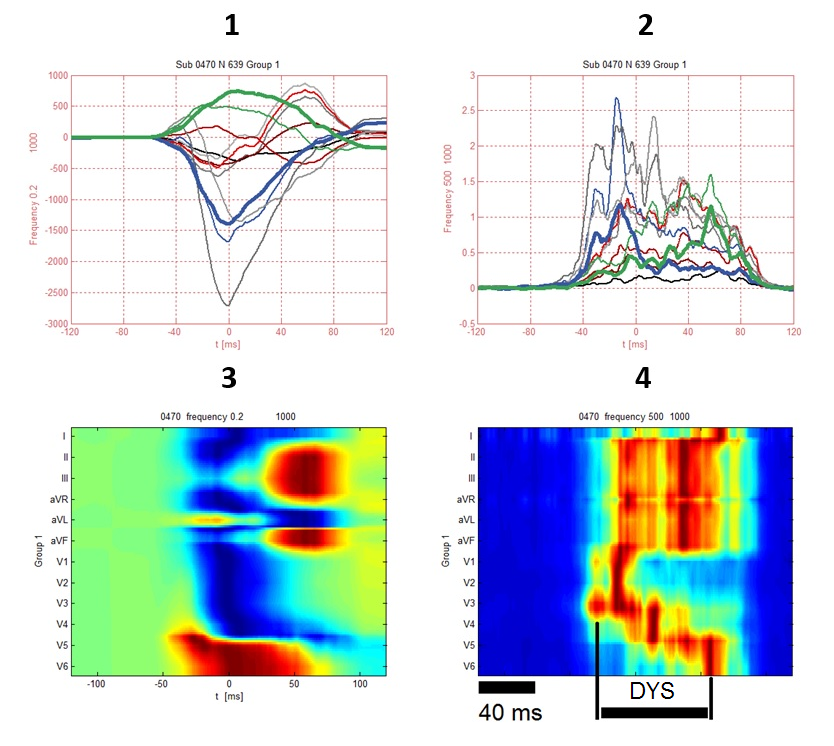
**

*Figure S7C: Averaged QRS complexes and maps - 12-lead ECG representation.*

*1: averaged QRS complexes, 12-lead ECG, 2: UHFQRS, 3: normalized map of QRS in frequency band 0.2-1000 Hz, 4: normalized UHFQRS map in frequency band 500-1000 Hz. The UHFQRS morphology is identical in I, II, III, aVR, aVL and aVF leads (4). The UHF dyssynchrony information is present in V leads only. In contrast, the shape of the QRS complex changes in all leads (3). This represents the difference between ECG and UHFQRS. LBBB can be determined precisely from the distribution of UHF components predominantly in V1, V2 and V5, V6 leads. Moreover, the time delay in milliseconds can be detected very accurately; the DYS parameter defines 78 ms maximal dyssynchrony (4). This is not possible in any standard QRS complex and also in UHF components of I, II, III, aVL, aVR and aVF leads.*

*
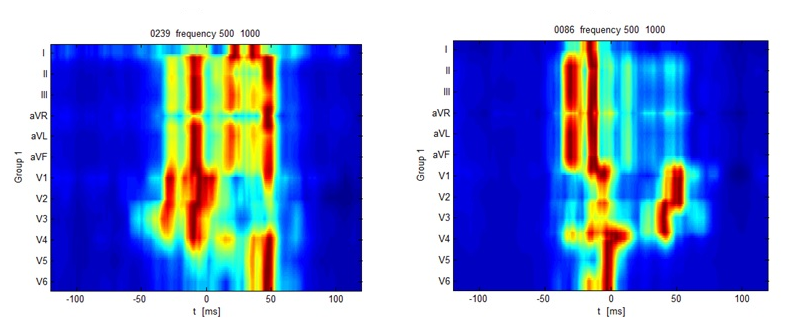
*

*Figure S7D: Additional examples of normalized 12-lead ECG maps of UHFQRS.*

*Left panel: LBBB, Right panel: RBBB patient.*

**CRT effect on UHFQRS and 6-month reverse remodeling with cardiac resynchronization.**

The following Figure S8 introduces examples of 7 CRT patients and 4 healthy subjects. Hemodynamic parameters were measured by MRI before CRT implantation and after 6-month follow-up with Bi-ventricular pacemaker in MRI safe mode (CRT function-OFF). In each subject, upper panel includes V1 and V6 UHFQRS before CRT implantation and bottom panel after 6-month follow-up with CRT ON. Patients 1-6 evince positive LV remodeling effect on cardiac function - increase in LVEF and decrease in ESV after 6-months of CRT.


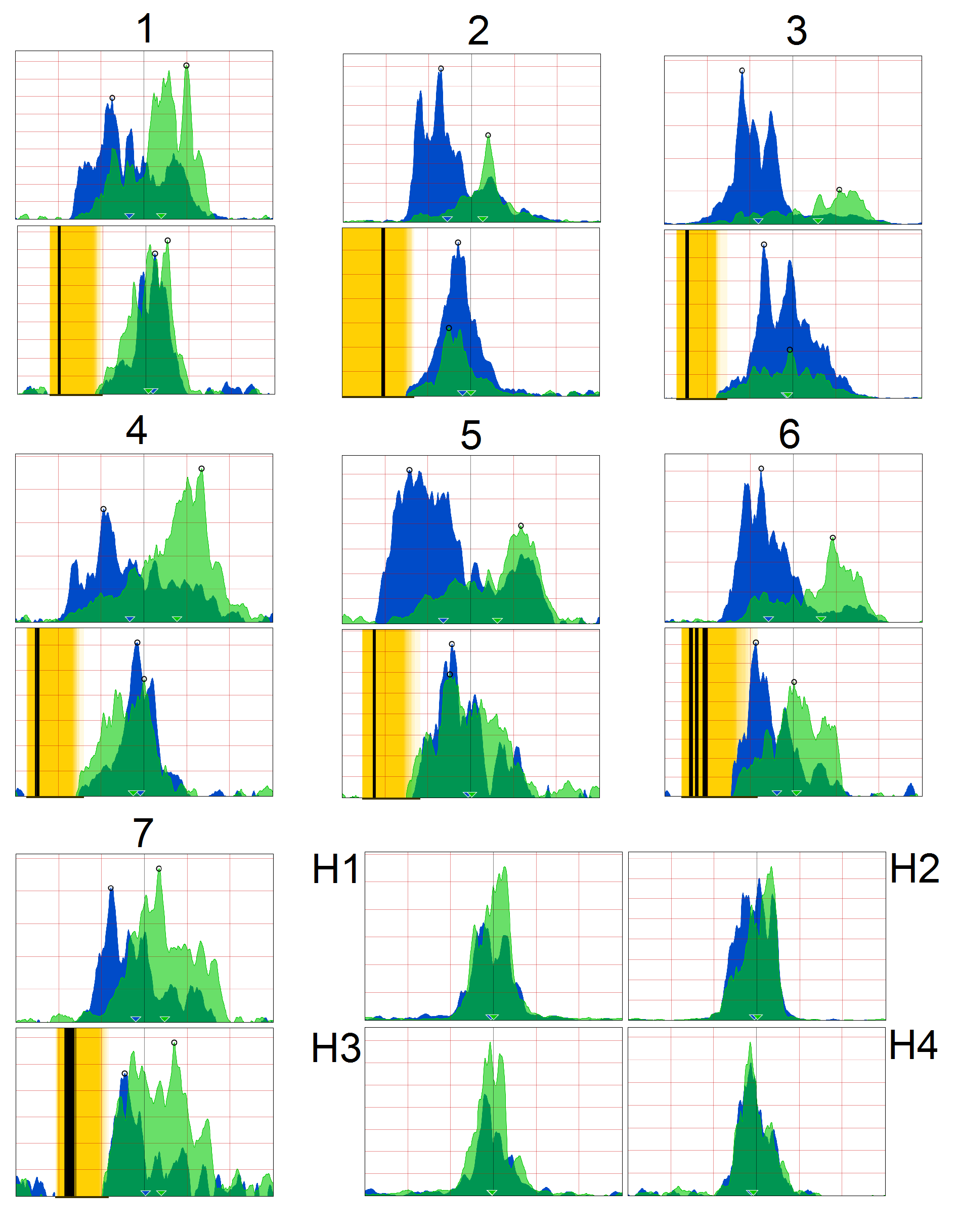


*Figure S8:*

*Each panel includes UHFQRS computed from V1 and V6 leads before CRT and after 6 months during CRT. DCM - dilated cardiomyopathy, LBBB – left bundle branch block, CAD – coronary artery disease, ESV – left ventricular end-systolic volume, LVEF – left ventricular ejection fraction,*

*Patient 1 – female, 62 years old, DCM, LBBB, ESV 118ml, LVEF 41 %, 6-month follow-up with CRT: ESV 41ml, LVEF 48 %*

*Patient 2 – male, 77, CAD, LBBB, ESV 92 ml, LVEF 35 %, 6-month follow-up with CRT:ESV 47 ml, LVEF 60 %*

*Patient 3 – male, 68, CAD, LBBB, ESV 271 ml, LVEF 21 %, 6-month follow-up with CRT:ESV 171 ml, LVEF 27 %*

*Patient 4 – male, 66, DCM, LBBB, ESV 101 ml, LVEF 34 %, 6-month follow-up with CRT: ESV 48 ml, LVEF 60 %*

*Patient 5 – female, 63, CAD,LBBB, ESV 128 ml, LVEF 22 %, 6-month follow-up with CRT: ESV 71 ml, LVEF 52 %*

*Patient 6 – female, 61, DCM, LBBB, ESV 77 ml, LVEF 35 %, 6-month follow-up with CRT: ESV 31 ml, LVEF 63 %,*

*Patient 7 - male, 73, DCM+CAD, LBBB, ESV 34 ml, LVEF 58 %, 6-month follow-up with CRT: ESV 52 ml, LVEF 57 %,*

*Healthy subjects: H1 – male, 50, H2 – male, 31, H3 – female, 69, H4 – female, 68*
